# Effects of TcFLA‐1BP and TcGP72 Deletion on the Infectivity and Survival of *Trypanosoma cruzi* in Cell Cultures

**DOI:** 10.1002/cbin.70076

**Published:** 2025-09-03

**Authors:** Normanda Souza‐Melo, Giovanna Henriques de Souza, Wanderley de Souza

**Affiliations:** ^1^ Laboratório de Ultraestrutura Celular Hertha Meyer, Centro de Pesquisas em Medicina de Precisão Instituto de Biofísica Carlos Chagas Filho— Universidade Federal do Rio de Janeiro, Instituto Nacional de Ciência e Tecnologia em Biologia Estrutural e Biomagens, Centro Nacional de Biologia Estrutural e Bioimagens, Universidade Federal do Rio de Janeiro Rio de Janeiro Brazil; ^2^ Av. Carlos Chagas Filho, 373, Centro de Ciências da Saúde Cidade Universitária, Ilha do Fundão, Rio de Janeiro Rio de Janeiro Brazil; ^3^ Centro de Estudos Biomédicos‐CMABio, Escola Superior de Saúde Universidade do Estado do Amazonas‐UEA, Manaus, Av. Carvalho Leal Manaus Brazil

**Keywords:** biological cycle, cytoskeletal remodulation, FAZ (flagellum attachment zone), TcFLA‐1BP, TcGP72, *Trypanosoma cruzi*

## Abstract

Chagas disease, caused by the protozoan *Trypanosoma cruzi*, is a neglected tropical disease with limited treatment options and no available vaccine. Understanding the role of proteins in the parasite's biological cycle is critical for advancing vaccine development and optimizing therapies. The flagellar attachment zone (FAZ) proteins play a pivotal role in motility, pathogenicity, and cell division in trypanosomatids, but their functions in *T. cruzi* are not as well‐characterized as in *Trypanosoma brucei* and *Leishmania spp*. This study investigates the orthologous TcGP72 and TcFLA‐1BP proteins in *T. cruzi*, focusing on their roles in the infective forms of the parasite. Our findings demonstrate that TcFLA‐1BP is important for efficient host cell infection in vitro, indicating its critical role in the parasite′s infectivity. Conversely, TcGP72 is nonessential for the infection process, but significantly contributes to cytoskeletal remodeling during the parasite′s life cycle. These results provide new insights into the distinct functional roles of FAZ proteins in *T. cruzi*. Furthermore, the study underscores the importance of TcGP72 in maintaining cellular architecture, reinforcing the relevance of FAZ proteins in the parasite′s pathogenesis and structural integrity.

## 1 Introduction

Chagas disease, caused by the protozoan *Trypanosoma cruzi*, poses a major public health threat, particularly in Latin America, but increasing globalization and migration have expanded its impact worldwide https://www.who.int/health-topics/chagas-disease#tab=tab_1. Although treatable in early stages, its variable symptomatology hampers early diagnosis, and about 30% of cases progress to chronic forms with severe cardiac and digestive complications, limiting treatment efficacy (Lee et al. 2013; Lidani et al. 2019). The lack of a widely available vaccine underscores the need for preventive strategies, emphasizing research on parasite virulence mechanisms and novel therapeutic targets.

*T. cruzi* alternates between different forms throughout its life cycle: epimastigotes and metacyclic trypomastigotes in the invertebrate host, and amastigotes and bloodstream trypomastigotes in the vertebrate host (De Souza 1984; Moretti et al. 2020; Zuma et al. 2021). Metacyclic trypomastigotes invade mammalian cells and differentiate into amastigotes, which replicate and later transition into infective trypomastigotes, perpetuating the infection cycle (Burleigh and Andrews 1995; De Souza 1984; Zuma et al. 2021). These morphological transitions involve extensive gene expression modulation and intracellular structural rearrangements, particularly in the flagellum and associated cytoskeletal elements (Vidal and Souza 2017; Zuma et al. 2021).

The dynamics of morphological alterations are driven by changes in gene expression, accompanied by rearrangements of intracellular structures in *T. cruzi*. The identification of the different forms of the parasite is dependent of several criteria as the relative positioning of some key organelles, including the nucleus, the kinetoplast, the structural organization of the kinetoplast DNA network, the emergence of the flagellum, and the position and length of the flagellum along the cell body (Avila et al. 2003; De Souza 1984; Gonçalves et al. 2018; Vidal and De Souza 2022). *T. cruzi* has a single flagellum responsible for motility and pathogenicity. It presents the classical 9 + 2 axonemal microtubule pattern but also contains a sophisticated network of filaments that form the paraflagellar rod (PFR). The flagellum of trypanosomatids is essential for parasite motility and acts as an anchoring platform for signaling complexes, influencing the dissemination of the parasite in host tissues (Ballesteros‐Rodea et al. 2012; Bastin et al. 1998; Hill 2003).

The topological organization of intracellular structures in trypanosomatids depends mainly on the arrangement of subpellicular microtubules, together with structures such as the flagellar adhesion zone (FAZ), which plays a fundamental role in directing the growth of cellular structures, being essential for morphogenesis, cell division, motility, and pathogenicity (Jesus et al. 1993; Halliday et al. 2020; Rotureau et al. 2014; Souza‐Melo et al. 2023; Vickerman 1969; Woodward et al. 1995).

The FAZ in the parasite presents similarities to the anchoring junctions found in epithelial cells of higher eukaryotes (Martínez‐Palomo et al. 1976; Nekrasova and Green 2013; Sunter and Gull 2016). In *T. cruzi*, the lateral attachment of the flagellum to the plasma membrane and the cytoskeleton of the cell body is mediated by the FAZ, which extends along the cell body, leaving a region of the flagellum free (De Souza 1999; Pimenta et al. 1989; Vidal and Souza 2017). This FAZ configuration resembles that found in *T. brucei* (Sun et al. 2013; Sunter and Gull 2016). In contrast, in Leishmania, the FAZ is restricted to the neck of the flagellar pocket, resulting in a free flagellum (Halliday et al. 2020). The FAZ is composed of four distinct domains: the flagellar FAZ domain, the intercellular FAZ domain, the FAZ filament domain, and the microtubule quartet binding domain (MtQ) (Sunter and Gull 2016). Previous freeze‐fracture studies have shown the presence of special intramembranous particles, which correspond to integral membrane proteins, in the fracture faces of the flagellar membrane and, in the case of trypomastigotes, also in the membrane lining the cell body (De Souza et al. 1978; Martínez‐Palomo et al. 1976). Although the structural organization of the FAZ of *T. cruzi* has been well characterized there are few information about its protein composition.

The interaction between the FLA‐1 and FLA1‐BP proteins, particularly in *T. brucei*, is one of the most well‐characterized aspects of the structural organization of the flagellar adhesion zone (FAZ). This interaction is crucial for anchoring the flagellum to the parasite′s cell body, playing a central role in FAZ assembly throughout the life cycle. Evidence indicates that FLA‐1 and FLA1‐BP possess transmembrane and extracellular domains, with their interaction occurring via their extracellular regions (LaCount et al. 2002; Souza‐Melo et al. 2023; Sun et al. 2018; Wheeler et al. 2019).

TcGP72 and TcFLA‐1BP interact within the intracellular region of the FAZ. However, their subcellular localizations reveal a species‐specific inversion in FAZ domain association between *T. brucei* and *T. cruzi*. In *T. brucei*, the TcGP72 homolog is predominantly localized to the cell body‐associated domain of the FAZ, while FLA‐1BP localizes to the flagellar‐associated domain. Conversely, in *T. cruzi*, TcGP72 is restricted to the flagellar FAZ domain, whereas TcFLA‐1BP is confined to the cell body FAZ domain, highlighting the divergent spatial organization of FAZ components across species (Souza‐Melo et al. 2023; Sun et al. 2013). The FLA1 protein in *T. brucei* and *Leishmania* exhibits homology to TcGP72 in *T. cruzi* (Halliday et al. 2019; Sunter and Gull 2016). Previous studies have demonstrated that deletion of these proteins results in alterations in cell growth, cytokinesis, and parasite morphometry, with more pronounced effects observed in the TcGP72^−/−^ mutant. Although the absence of FLA‐1 or FLA1‐BP in both parasites leads to morphologically aberrant forms, the complete disruption of the FAZ observed in the TcGP72^−/−^ mutant directly impacts metacyclogenesis and the rearrangement of intracellular organelles. This phenotype is less severe in TcFLA‐1BP^−/−^ parasites, suggesting a differential contribution of these proteins to the maintenance of the structural and functional integrity of the FAZ, as observed in both epimastigote and metacyclic trypomastigote forms (Jesus et al. 1993; Souza‐Melo et al. 2023).

Given the roles of the TcGP72 and TcFLA‐1BP proteins in cell growth, division, differentiation during metacyclogenesis, and organelle repositioning, we propose that these proteins play a significant role in the *T. cruzi* biological cycle within the mammalian host. In this study, we characterized the functions of TcGP72 and TcFLA‐1BP during the infective stages of the parasite, assessing their impact on differentiation capacity, organelle remodeling, and infection progression using two independent mutant lines—one with a double deletion of the TcGP72 gene, and another with a double deletion of the TcFLA‐1BP gene.

## 2 Results

### 2.1 Stable Deletion of TcFLA‐1BP and TcGP72 Genes in Trypomastigotes Forms

To confirm that the gene deletions persisted in the trypomastigote stage of the TcFLA‐1BP and TcGP72 knockout parasites, the absence of both genes was verified by PCR (Figure S1). The trypomastigotes analyzed were obtained by infecting mammalian cells with metacyclic trypomastigotes derived from epimastigote knockout lines for TcFLA‐1BP and TcGP72. The absence of these proteins in epimastigotes had previously been demonstrated in these lines (Souza‐Melo et al. 2023). Primers P1‐P2 amplified the coding regions of the genes of interest, while primers P1‐P5 amplified a region including the integration site. As shown in Figure S1B, the absence of the expected 737 bp (TcGP72) and 654 bp (TcFLA‐1BP) bands in the knockout parasites, amplified by primers P1–P2, verified the complete deletion of the genes. The presence of bands in PCRs with primers P1–P5 in these parasites but not in the T7 control, corresponding to the blasticidin and hygromycin resistance cassettes, indicated the correct integration of the cassettes at the deletion sites. These results demonstrate the persistence of the TcFLA‐1BP and TcGP72 gene absence in cell culture‐derived trypomastigote forms of the knockout lines.

### 2.2 The Role of TcFLA‐1BP and TcGP72 in Maintaining Flagellar Integrity in Infective Forms of *Trypanosoma cruzi*

**Figure 1:**
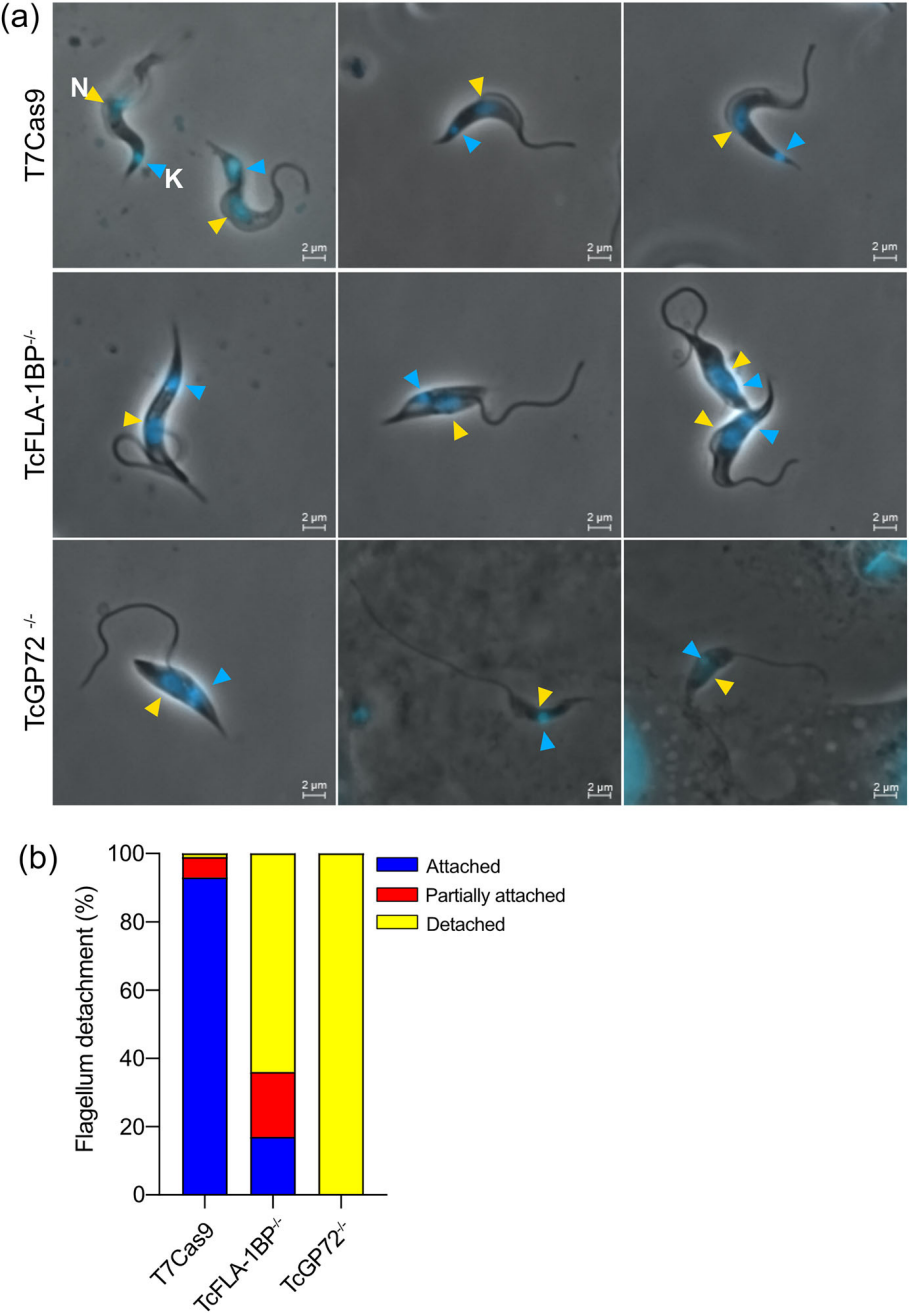
Analysis of flagellar adhesion in *Trypanosoma cruzi* trypomastigotes by fluorescence microscopy. (a) Fluorescence microscopy images of T7Cas9 (control), TcFLA‐1BP^−/−^, and TcGP72^−/−^ trypomastigotes stained with Hoechst. N (yellow arrowhead): nucleus; K (blue arrowhead): kinetoplast. Scale bar: 2 μM. (b) Quantification of flagellar adhesion in trypomastigotes (*n* = 300).

To investigate the role of TcFLA‐1BP and TcGP72 proteins in preserving the structural integrity of the parasite *T. cruzi*, we evaluated flagellar adhesion in trypomastigote forms (Figure 1) LLC‐MK2 cells were infected with metacyclic trypomastigotes, and the released culture‐derived trypomastigotes (TCTs) were collected and stained with Hoechst to visualize the nucleus and kinetoplast. Fluorescence microscopy analysis revealed significant differences in cell morphology among the analyzed lineages. In the control lineage (T7Cas9), most parasites exhibited the classical trypomastigote morphology, with the flagellum firmly attached to the cell body, an elongated central nucleus, and a posteriorly positioned kinetoplast. In contrast, in the TcFLA‐1BP^−/−^ and TcGP72^−/−^ knockout lineages, there was a substantial increase in the frequency of parasites with partially or completely detached flagella. In the TcFLA‐1BP^−/−^ lineage, 19% of parasites had partially attached flagella, 64% exhibited completely detached flagella, and only 17% maintained flagellar adhesion. The TcGP72^−/−^ lineage presented an even more homogeneous phenotype with 100% of parasites exhibiting complete flagellar detachment, suggesting a generalized collapse of the FAZ structure.

**Figure 2:**
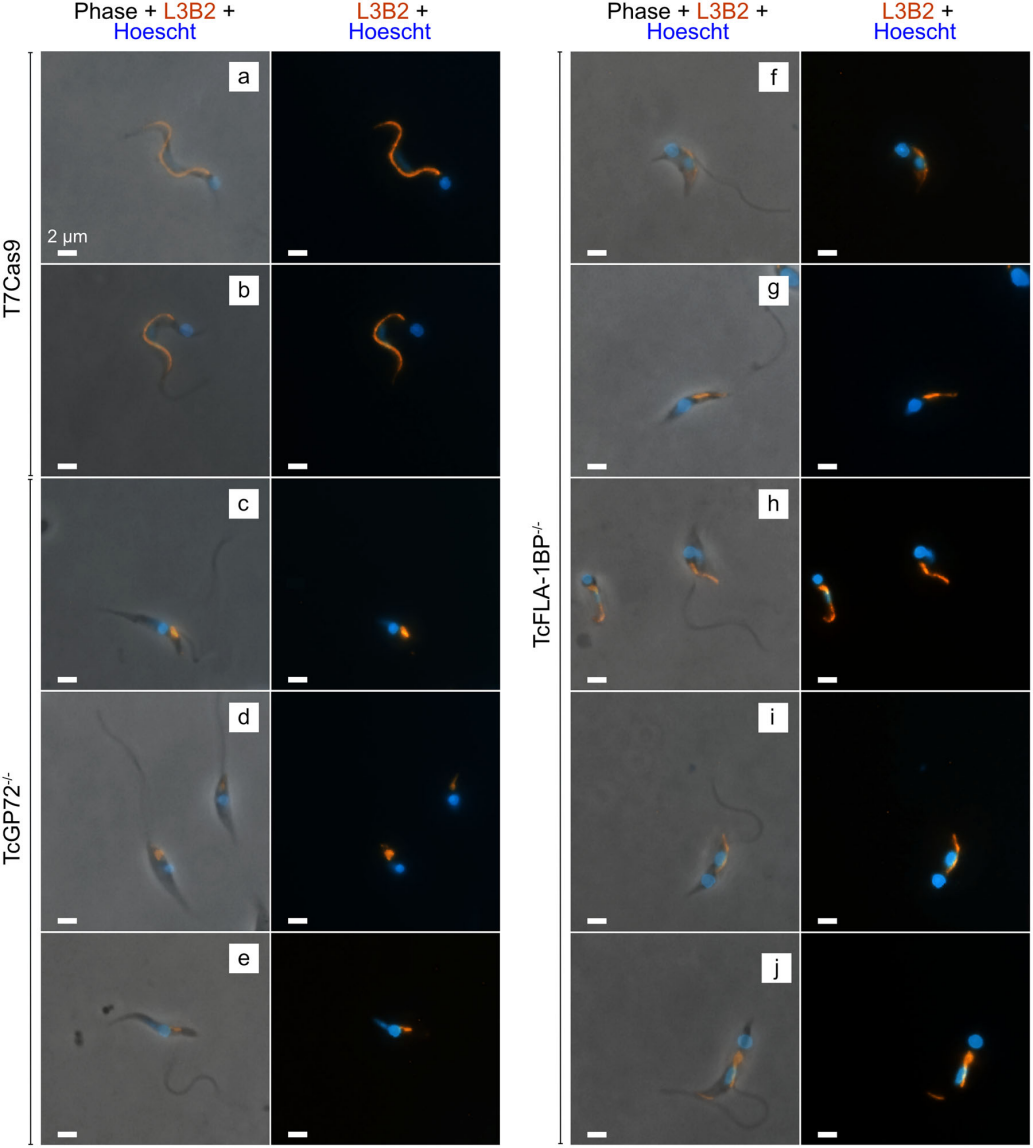
Impact of TcFLA‐1BP and TcGP72 protein deletions on flagellar attachment zone (FAZ) assembly. Fluorescence microscopy of tissue culture‐derived trypomastigotes. The FAZ was labeled with anti‐FAZ (L3B2 antibody) [red]. Nuclei and kinetoplasts are stained with Hoechst (blue). T7Cas9 (control): (a, b) TcGP72^−/−^: (c–e) TcFLA‐1BP^−/−^: (f–j) Scale bar: 2 μM.

Intending to investigate the disruption of the FAZ in TCTs, we performed an immunofluorescence analysis using the L3B2 antibody, which recognizes the FAZ‐1 protein (Figure 2). The results obtained reveal that mutations in the TcFLA‐1BP and TcGP72 proteins result in a significant disorganization of the FAZ, although acting distinctly. In the control parasite T7Cas9 (Figure 2a,b), the FAZ staining is continuous along the cell body, indicating structural integrity. In the TcFLA‐1BP^−/−^ mutant, the FAZ exhibits an irregular staining pattern, but we observed that, even with flagellar detachment, the staining in the cell body remains. This staining seems to depend on the degree of flagellar detachment: parasites with a completely detached flagellum show continuous staining along the cell body (Figure 2f–i), while those with partial detachment exhibit interruptions in the staining (Figure 2j). In TcGP72^−/−^ mutants (Figure 2c–e), with a completely detached flagellum, L3B2 staining accumulates near the flagellar pocket at the base of the flagellum.

### 2.3 TcFLA‐1BP and TcGP72 Deletions Induce Significant Ultrastructural Changes in *Trypanosoma cruzi* Trypomastigotes

**Figure 3:**
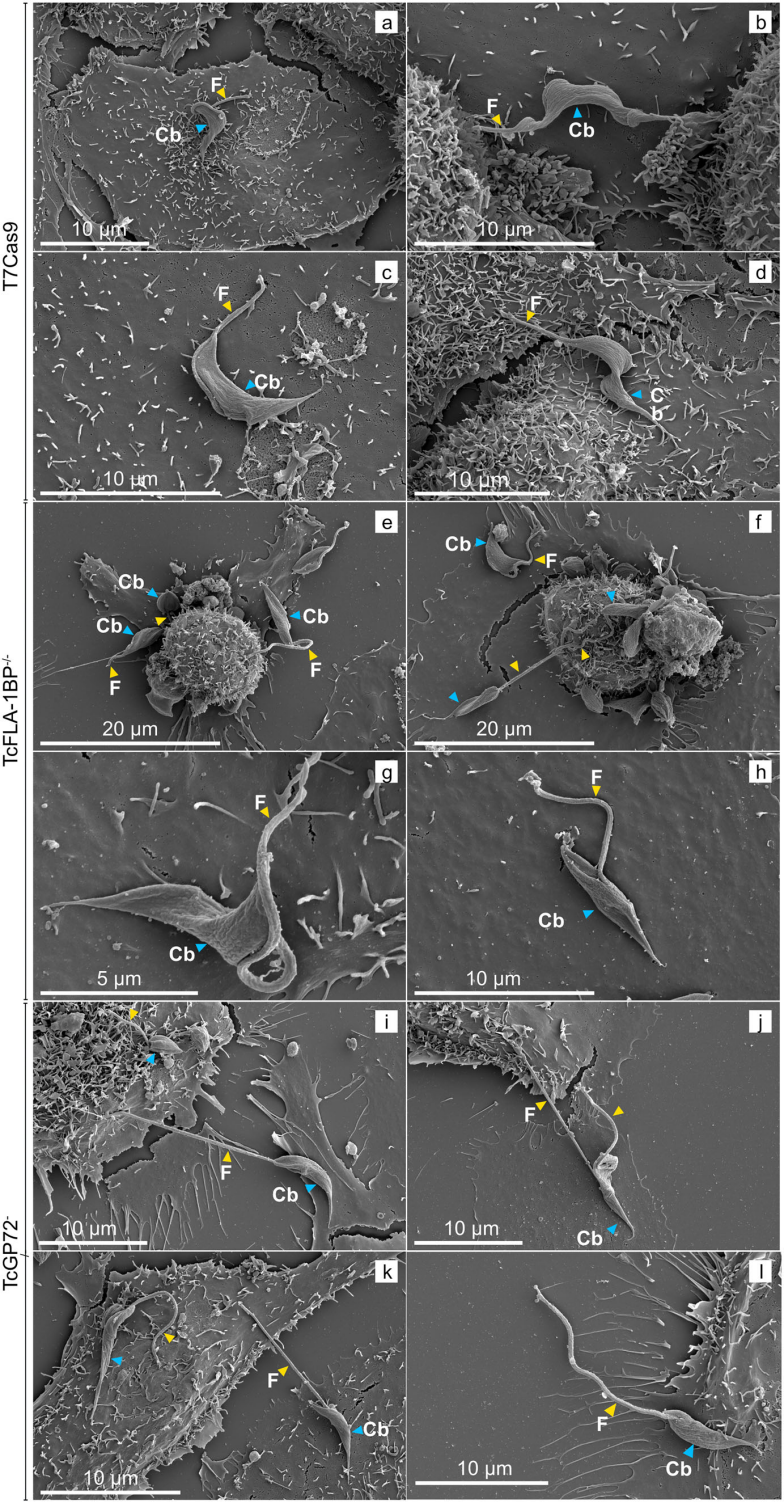
Scanning electron microscopy (SEM) of *Trypanosoma cruzi* trypomastigotes adhered to LLC‐MK2 cells. (a–d) depict wild‐type T7Cas9 trypomastigotes showing the characteristic elongated body and flagellum (f, yellow arrowhead) with normal attachment to the host cells via the cell body (Cb, blue arrowhead). (e–h) show TcFLA‐1BP^−/−^ and (i–l) TcGP72^−/−^ knockout mutants, respectively, with altered morphology. The mutants exhibit abnormal curvature and a flagellum that is either partially or completely detached from the cell body, showing structural differences compared to the control.

Scanning electron microscopy (SEM) was employed to thoroughly investigate the ultrastructural alterations resulting from the deletion of the TcFLA‐1BP and TcGP72 genes in the trypomastigote forms of *Trypanosoma cruzi* (Figure 3), compared to the T7Cas9 control strain. The T7Cas9 control strain exhibited the typical trypomastigote morphology, characterized by a flagellum firmly attached to the cell body along the entire FAZ, and a fusiform cell body with tapered ends (Figure 3a–d). In the TcFLA‐1BP^−/−^ mutant strains, a phenotype characterized by flagellar detachment was observed, ranging from partial (Figure 3f,g) to complete detachment (Figure 3f–h). Forms with partially detached flagella maintained a morphology similar to the control, with an elongated cell body and tapered ends. However, in forms with completely detached flagella, the cell body exhibited a fusiform structure with tapered ends (Figure 3h), or one end slightly thinner than the other (Figure 3e,f), resembling the promastigote morphology of *Leishmania*. This promastigote‐like phenotype was more pronounced in the TcGP72^−/−^ strains, where 100% of the parasites showed a completely detached flagellum from the cell body.

### 2.4 The Influence of TcFLA‐1BP and TcGP72 Deletion on Surface Protein Distribution in *Trypanosoma cruzi* Trypomastigotes

To assess the molecular changes in the trypomastigote forms of *T. cruzi* T7Cas9 and mutants lacking the TcFLA‐1BP and TcGP72 proteins, we immunolocalized trans‐sialidase, a protein specific to the trypomastigote stage, using anti‐trans‐sialidase antibodies (Mab39). Trans‐sialidase was detected by immunofluorescence in all the parasites; however, distinct labeling patterns were observed between the groups (Figure S2).

In the T7Cas9 parasites, the trans‐sialidase was mainly distributed along the cell body, with diffuse and dotted labeling on the cell surface, and no significant labeling was detected in the flagellum (Figure S2a,b). This pattern is compatible with the expected localization of trans‐sialidase in trypomastigote forms of *T. cruzi*, associated with the cell surface. In contrast, the mutants lacking TcFLA‐1BP and TcGP72 showed altered immunofluorescence patterns. In the TcGP72^−/−^ mutant (Figure S2c–e), the trans‐sialidase showed a more intense point distribution along the cell body and flagellum, with labeling points scattered throughout the flagellar structure. The TcFLA‐1BP^−/−^ mutant (Figure S2f–h) exhibited diffuse and punctate labeling similar to that observed in TcGP72^−/−^, but with reduced intensity, probably due to the morphological heterogeneity of the culture.

### 2.5 TcFLA‐1BP Deficiency Impairs *Trypanosoma cruzi* Infection in LLC‐MK2 Cells

The altered localization of trans‐sialidase and the morphological changes observed in the TcFLA‐1BP^−/−^ and TcGP72^−/−^ mutants underscore the critical role of TcFLA‐1BP and TcGP72 in maintaining the structural organization and proper distribution of key surface molecules in *Trypanosoma cruzi*. Given the importance of morphological alterations and surface proteins, such as trans‐sialidase, in host‐cell interactions and parasite infectivity, we further explored how these modifications affect the ability of *T. cruzi* to invade host cells. We conducted infectivity assays using mammalian host cells to investigate whether defects in the morphology and distribution of surface proteins impact parasite infectivity. In the infectivity assays, we evaluated the following parameters: adhesion rate, percentage of infected cells, infection index, average number of parasites per infected cell, multiplication rate, and egress.

To determine if the morphological alterations observed in the mutants affected the adhesion capacity, infection assays were performed using LLC‐MK2 cells. After 1 h of incubation, the non‐internalized parasites were removed, and the remaining attached parasites were quantified (Figure S3). The results showed that the TcFLA‐1BP^−/−^ strain exhibited a statistically significant reduction (*p* = 0.0489) in the number of parasites attached per cell, indicating a crucial role of the TcFLA‐1BP protein in mediating the initial events of parasite‐host interaction. The TcGP72^−/−^ strain did not show a significant difference in adhesion compared to the T7Cas9 control.

To assess other infection parameters, including the percentage of infected cells, infection index, average number of parasites per infected cell, and intracellular multiplication rate, we conducted extended infection assays with incubation times of 4, 24, and 48 h. Parasites were initially incubated with LLC‐MK2 cells for 4 h to allow their internalization. Non‐internalized parasites were subsequently removed, and cells were re‐incubated for 24 and 48 h to enable intracellular parasite replication. Following incubation, cells were fixed, stained, and analyzed to quantify infection rates, infection indices, and the number of amastigotes per cell. The infection index, reflects both the extent of host cell infection and the parasite load, providing an integrated measure of infection efficiency.

**Figure 4:**
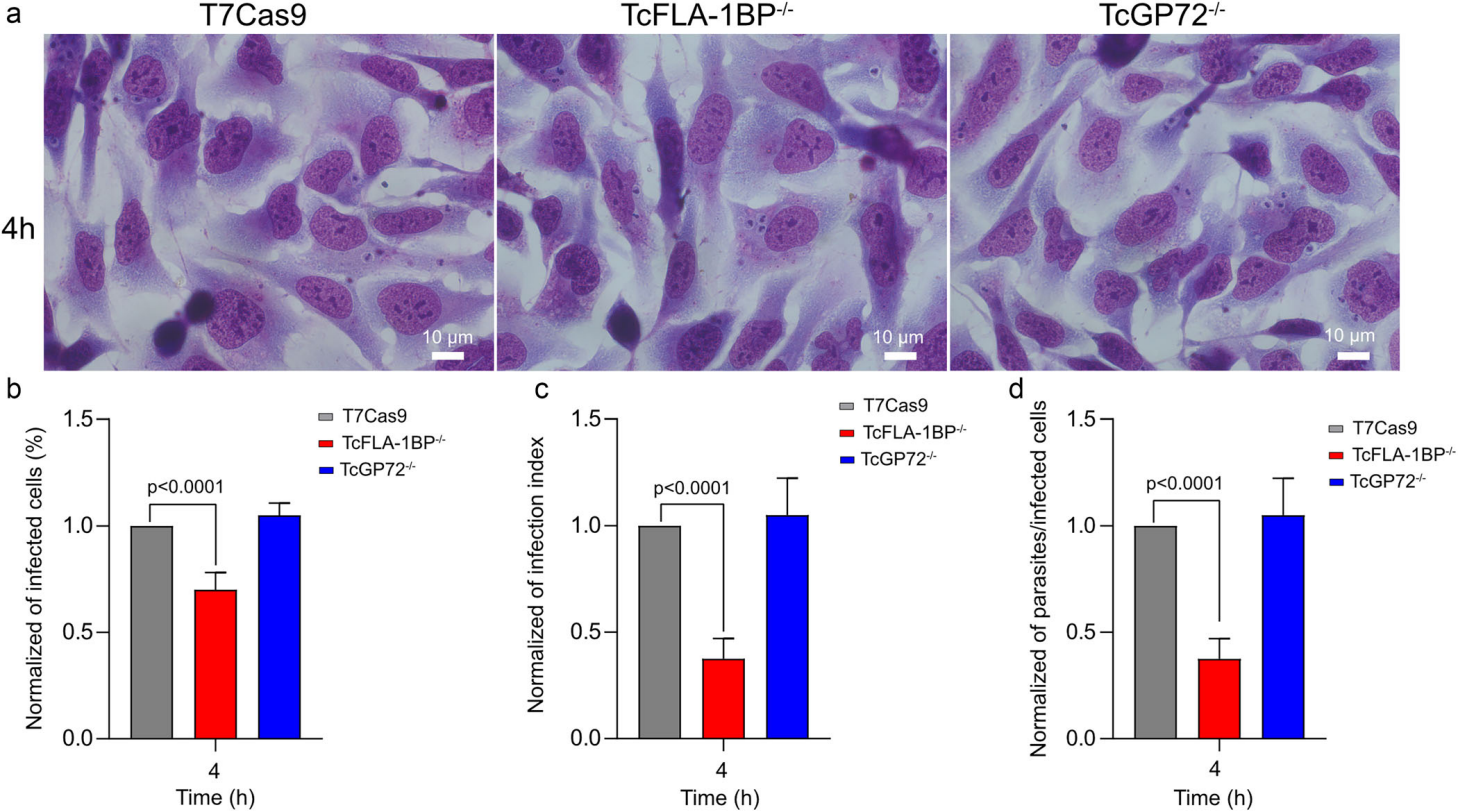
Analysis of trypomastigote infection efficiency in LLC‐MK2 cells. (a) Representative optical microscopy images of LLC‐MK2 cells infected with trypomastigotes from the T7Cas9 (control), TcFLA‐1BP^−/−^, and TcGP72^−/−^ strains after 4 h of incubation. The scale bar represents 10 μM. (b–d) Graphs representing the percentage of infected cells (%iC), infection index (IntP/TotalC), and number of intracellular amastigotes per LLC‐MK2 cell, respectively. Data were normalized and represented the mean ± standard deviation from four independent experiments. Statistical significance was determined using one‐way ANOVA with Tukey′s multiple comparisons test. *p* < 0.001 versus control. Total number of cells = 300.

**Figure 5:**
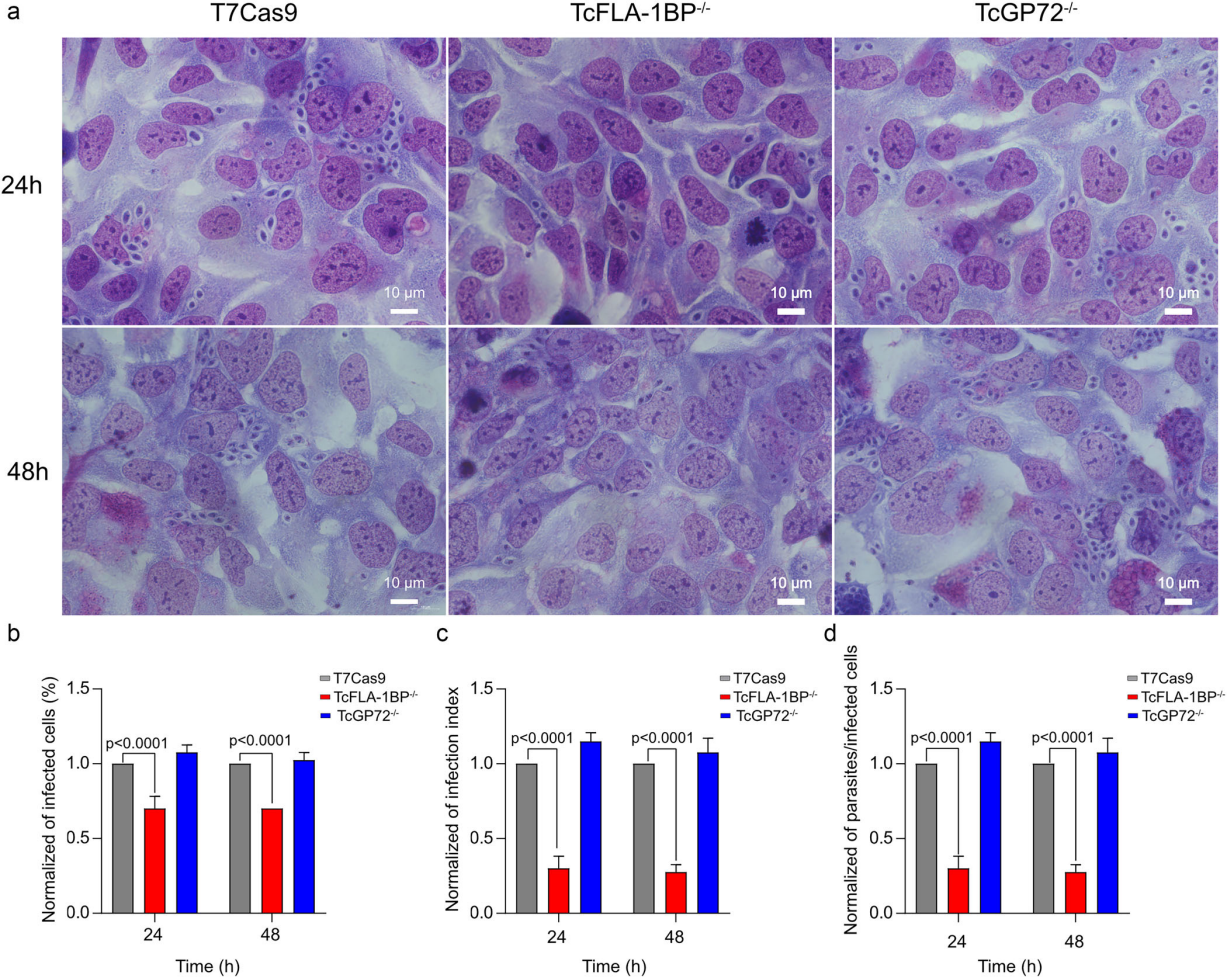
Evaluation of trypomastigote multiplication and infection dynamics in LLC‐MK2 cells. (a) Representative optical microscopy images of LLC‐MK2 cells infected with trypomastigotes from the T7Cas9 (control), TcFLA‐1BP^−/−^, and TcGP72^−/−^ strains after 24 and 48 h of incubation. The scale bar represents 10 μM. (b–d) Graphs depicting the percentage of infected cells (%iC), infection index (IntP/TotalC), and number of intracellular amastigotes per LLC‐MK2 cell, respectively. Data are normalized and represent the mean ± standard deviation from four independent experiments. Statistical significance was determined using one‐way ANOVA with Tukey′s multiple comparisons test. *p* < 0.001 versus control. Total number of cells = 300.

The results obtained at short infection times (4 h) demonstrate the crucial role of the TcFLA‐1BP protein (Figure 4). Deletion of the TcFLA‐1BP gene resulted in a significant reduction in all evaluated infection parameters, including the percentage of infected cells (~ 0.75%), infection index (~ 0.4), and number of intracellular parasites (~ 0.4) compared to T7Cas9. To gain a deeper understanding of the roles of TcFLA‐1BP and TcGP72 in the internalization and replication processes of *T. cruzi*, we extended the infection analysis beyond the initial 4‐h time point to include evaluations at 24 and 48 h. These extended time points provided critical insights into infection dynamics, focusing on the percentage of infected cells, the number of intracellular amastigotes per cell (Figure 5), and multiplication rates (Figure S4).

At 24 and 48 h, the analysis revealed a significant reduction in infection rates and intracellular parasite load in the TcFLA‐1BP^−/−^ mutant compared to the control (T7Cas9) and TcGP72^−/−^ strains. Figure 5A shows representative optical microscopy images of LLC‐MK2 cells infected with trypomastigotes from the T7Cas9 control. The TcFLA‐1BP^−/−^ mutant exhibited a sharp decline in infection, with infection rates of approximately 0.7% at both 24 and 48 h (Figure 5B). In contrast, the TcGP72^−/−^ knockout parasite displayed infection rates comparable to those of the T7Cas9 control.

Regarding the infection index (Figure 5C), the TcFLA‐1BP^−/−^ mutant demonstrated the most significant reductions, with infection indices of approximately 0.3 at both 24 and 48 h. Correspondingly, the number of intracellular parasites (Figure 5D) in this mutant was markedly lower, averaging around 0.3 at both time points. In comparison, the T7Cas9 control and TcGP72^−/−^ parasites exhibited similar infection indices across all time points evaluated.

We assessed the parasite multiplication rates at 24 and 48 h, and notable differences were observed among the strains (Figure S4). The TcFLA‐1BP^−/−^ mutant strain demonstrated a significantly longer multiplication time (~ 20 h) compared to the T7Cas9 control strain, which showed a multiplication time of around 16 h. In contrast, the TcGP72^−/−^ mutant strain did not exhibit significant differences in its multiplication rate when compared to the control. Notably, the infection patterns observed at 24 and 48 h were similar to those observed after 4 h of incubation for both knockout lines, indicating a consistent phenotype across time points.

### 2.6 Egress Dynamics of *Trypanosoma cruzi* Null Mutants in LLC‐MK2 Cells

**Figure 6:**
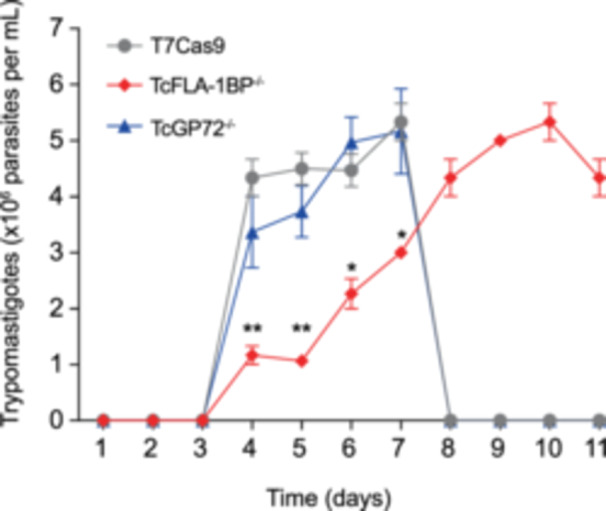
Trypomastigote release dynamics from LLC‐MK2 cells infected with *Trypanosoma cruzi* strains. The graph depicts the time course analysis of trypomastigote release into the supernatant following infection of LLC‐MK2 cells with T7Cas9 (control), TcFLA‐1BP^−/−^, and TcGP72^−/−^ trypomastigotes. Trypomastigote numbers were determined at indicated time points up to 11 days postinfection. Data are represented as mean ± standard deviation from three independent experiments. Statistical significance was determined using two‐way ANOVA with Tukey′s multiple comparisons test. *p < 0.05, **p < 0.001 versus control.

We examined the egress dynamics of *Trypanosoma cruzi* null mutants lacking the TcFLA‐1BP and TcGP72 proteins in LLC‐MK2 cells. Trypomastigotes from the T7Cas9 control strain and the TcFLA‐1BP and TcGP72 null mutants were used to infect LLC‐MK2 host cells, and the cultures were monitored over an 11‐day period to assess parasite release. Parasite numbers were quantified at various time points using a Neubauer chamber (Figure 6).

The TcFLA‐1BP^−/−^ mutant showed a significantly delayed egress pattern, with peak parasite release (5 × 106 parasites/mL) occurring on day 9, and the infection persists throughout the 11 days. In contrast, both the control T7Cas9 strain and the TcGP72^−/−^ mutant reached their peak egress between Days 6 and 7, followed by complete host cell lysis shortly after. On the other hand, the TcGP72^−/−^ mutant displayed egress dynamics similar to the control.

### 2.7 The Role of TcGP72 in Maintaining the Ultrastructural Integrity of *Trypanosoma cruzi* Trypomastigotes

To investigate the impact of the deletion of the TcFLA‐1BP and TcGP72 genes on the ultrastructure of *Trypanosoma cruzi* trypomastigote forms, we performed transmission electron microscopy (TEM) analyses on the T7Cas9 (control), TcFLA‐1BP^−/−^, and TcGP72^−/−^ strains. Our analysis focused on the detailed architecture, morphology, and spatial organization of key organelles, including the nucleus, kinetoplast, Golgi complex, and flagellum, revealing significant alterations.

**Figure 7:**
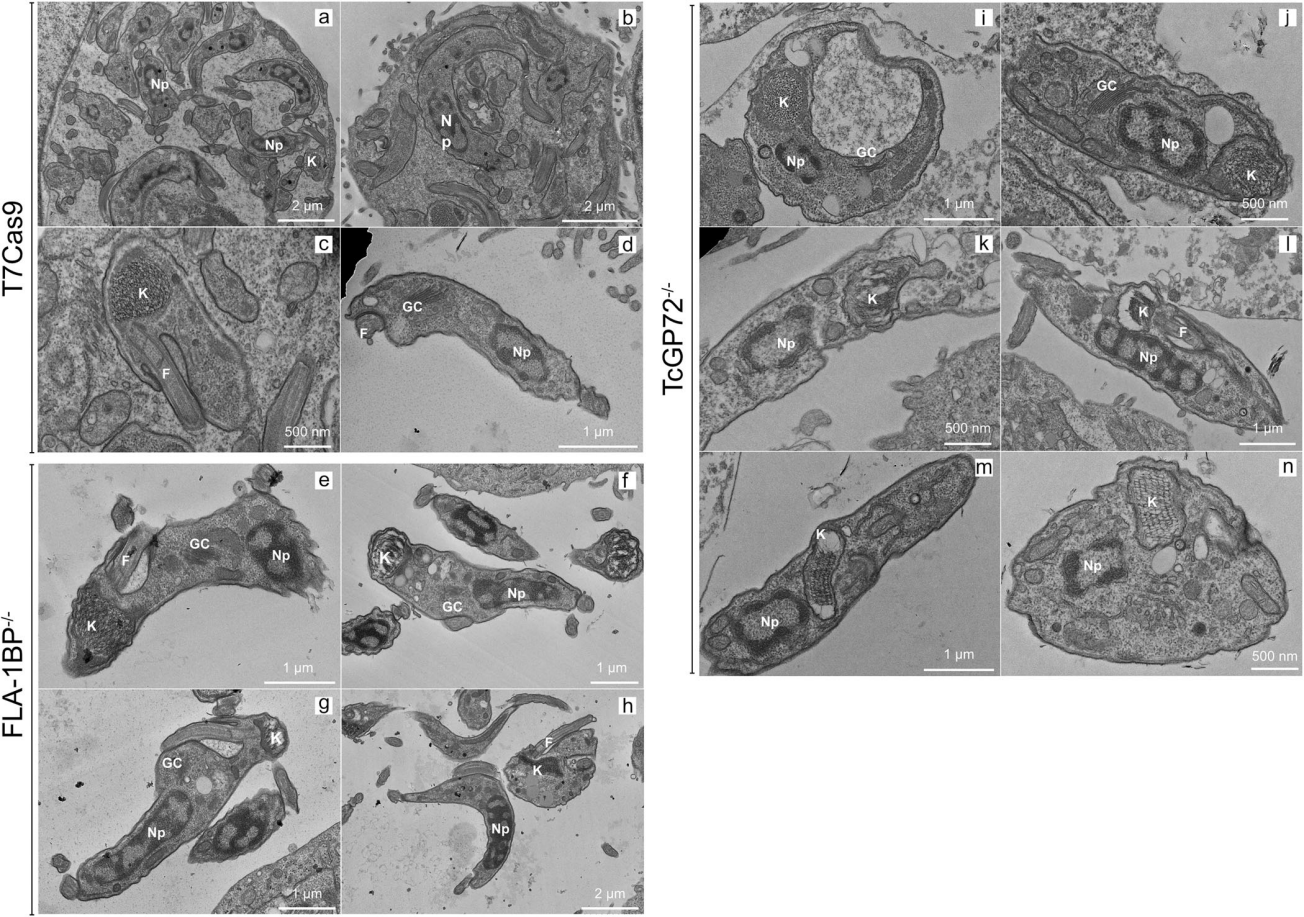
Ultrastructural analysis of *Trypanosoma cruzi* trypomastigotes from TcFLA‐1BP and TcGP72 knockout mutants. Transmission electron microscopy (TEM) images of *Trypanosoma cruzi* trypomastigotes infecting LLC‐MK2 cells. (a–d) T7Cas9 (control); (e–h) TcFLA‐1BP^−/−^; (i–n) TcGP72^−/−^. F, flagellum; GC, Golgi complex; K, kinetoplast; Np, parasite nucleus; Nc, cell nucleus; Nu, nucleolus.

Transmission electron microscopy (TEM) images of the trypomastigote forms revealed that the T7Cas9 control exhibited canonical trypomastigote ultrastructural features (Figure 7a–d). The nucleus was elongated and centrally located, with heterochromatin occupying the majority of the nuclear matrix and a discernible nucleolus absent. The kinetoplast was positioned posteriorly, displaying a rounded morphology with an irregularly arranged kDNA network. The flagellum emerged from the posterior region, and the Golgi apparatus was situated between the nucleus and the kinetoplast.

Microscopic analyses revealed that trypomastigote forms of the TcFLA‐1BP^−/−^ mutant exhibited, for the most part, the typical ultrastructural characteristics of trypomastigotes when compared to the T7Cas9 control (Figure 7e–h). However, the TcGP72^−/−^ mutant displayed significant ultrastructural alterations, notably a reorganization of the kDNA network and a displacement of organelles such as the kinetoplast and the Golgi complex (Figure 7i–n).

However, trypomastigotes of the TcGP72^−/−^ mutant exhibited alterations in nuclear morphology, presenting both elongated and globular structures, although heterochromatin occupied most of the matrix and no nucleolus was observed. The most significant alterations were observed in the structure of the kDNA network, which showed decompacted fibers (Figure 7k,m), fibers with intermediate decompaction with the antipodal sites showing a higher degree of decompaction but adhered to the mitochondrial membrane, and compacted fibers forming a kinetoplast with well‐defined layers (Figure 7n). Regarding topology, it was difficult to determine whether the kinetoplast was located in the posterior or anterior region due to flagellar detachment. However, in some images, we observed a location in the center of the cell body near the nucleus, a characteristic location of intermediate forms. A particularly noteworthy observation was that, despite the absence of positional rearrangement of the kDNA, the network underwent a process of decompaction. These findings suggest that the decompaction of the kDNA network and the topological rearrangement of the kinetoplast may be independent events, regulated by distinct mechanisms. In addition to the alterations observed in the kinetoplast, we noted a topological redistribution of the Golgi apparatus (Figure 7i–j). While in T7Cas9 trypomastigotes, the Golgi apparatus occupies a characteristic position between the kinetoplast and the nucleus, in TcGP72 knockout parasites, this organelle was found in a location opposite to the kinetoplast.

The observed alterations in the organization of the Golgi complex, kinetoplast, and kDNA network in TcGP72 mutant trypomastigotes demonstrate the central role of this protein in maintaining cellular architecture. As a fundamental component of the flagellar attachment zone (FAZ), TcGP72 acts as an orchestrator of the spatial organization of organelles, ensuring the structural integrity of the parasite.

### 2.8 Effects of TcFLA‐1BP and TcGP72 Deletion on the Subcellular Organization of *Trypanosoma cruzi* Amastigotes

**Figure 8:**
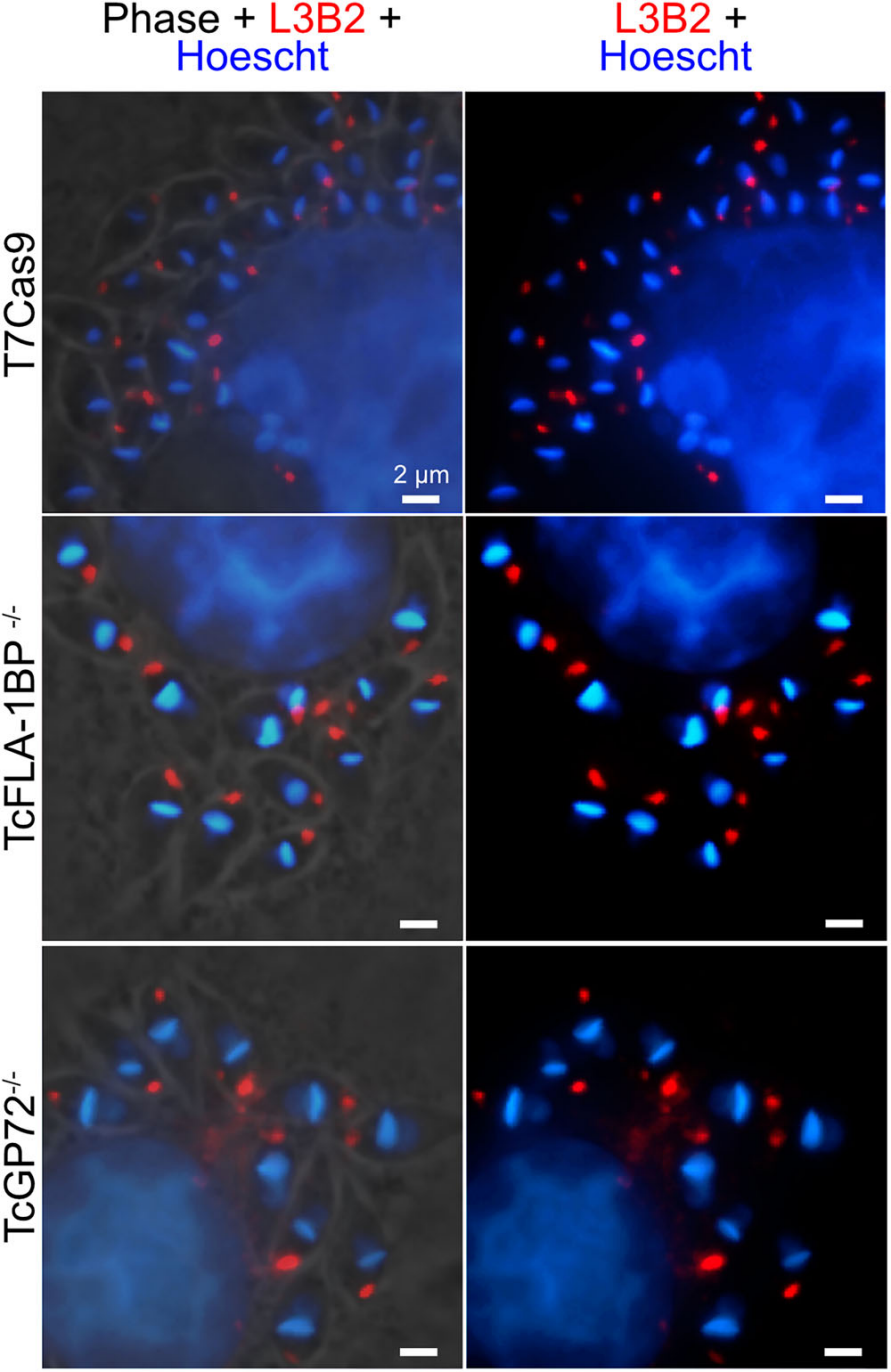
Localization of the flagellar attachment zone (FAZ) in *Trypanosoma cruzi* amastigotes. Immunofluorescence assay of *T. cruzi* amastigotes using the monoclonal antibody L3B2 (Anti‐FAZ‐1), which targets the parasite′s FAZ‐1 protein (red). Hoechst (blue) was used as a nuclear and kinetoplast stain. The images show the distribution of FAZ and the position of the nucleus and kinetoplast in T7Cas9 (control), TcGP72^−/−^, and TcFLA‐1BP^−/−^ amastigotes. Scale bars = 2 μM.

To assess whether the alterations observed in trypomastigotes extended to the amastigote stage, we performed immunofluorescence assays using the anti‐FAZ‐1 (L3B2) antibody in infected cells for 48 h (Figure 8). Analysis of FAZ distribution in amastigotes of TcFLA‐1BP and TcGP72 mutants revealed a localization pattern similar to that observed in the T7Cas9 control, with the protein concentrated in the region adjacent to the flagellar pocket. All parasites displayed the characteristic morphology of intermediate stage II amastigotes, with a short flagellum and a leaf‐shaped cell body.

In ultrastructural analyses of intracellular amastigote forms, we observed that both control and mutant parasites exhibited flagellum and kinetoplast at the anterior extremity (Figure S5a,b). When comparing the ultrastructures, we identified notable differences. In the T7Cas9 lineage, the nucleus displayed a rounded shape, with heterochromatin adjacent to the nuclear lamina, a central nucleolus, and a discoid kDNA network with condensed fibers. In TcFLA‐1BP knockout, in addition to this classical organization (Figure S5c), we observed unusual phenotypes, with contact between the mitochondrial membrane and the nuclear membrane, as well as the interaction between the mitochondrial membrane and the subcellular microtubule network (Figure S5e,f). In some parasites of this mutant, we also observed the presence of more than one nucleolus (Figure S5d). We believe that these unusual events, mainly observed in the TcFLA‐1BP^−/−^ mutant, may be related to the slowness of infection.

## 3 Discussion

This study examined the roles of TcFLA‐1BP and TcGP72 in *T. cruzi* development, revealing their essential functions in flagellar integrity, cell morphology, and infectivity. The FAZ, a specialized structure in trypanosomatids, shares organizational features with desmosomes in higher eukaryotes (Sunter and Gull 2016). Like desmosomes, which provide mechanical stability by linking cells via cadherins (Nekrasova and Green 2013), the FAZ anchors the flagellum to the cell body, ensuring proper motility, differentiation, and infection efficiency.

The TcGP72 and TcFLA‐1BP null mutants exhibited distinct phenotypes characterized by defects in flagellum adhesion, impaired FAZ assembly, alterations in protein transport, modifications in infectivity, and organelle rearrangement. The correlation between these varied phenotypes has already been documented, being related to the location of the proteins within the FAZ (Hayes et al. 2014; LaCount et al. 2002; Rocha et al. 2006; Sunter and Gull 2016; Vaughan et al. 2008; Zhou et al. 2015). Deletion of FAZ flagellar‐domain proteins typically leads to flagellum detachment and cell body reduction in *T. brucei* and *Leishmania* (Halliday et al. 2020), as observed in our epimastigote and metacyclic trypomastigote studies (Souza‐Melo et al. 2023). This phenotype predominated in TcGP72 knockouts, whereas TcFLA‐1BP deletion in the intracellular FAZ domain resulted in trypomastigotes with partially detached flagella, maintaining adhesion at the anterior tip.

Immunofluorescence data with the anti‐FAZ1 antibody (L3B2) and scanning electron microscopy in trypomastigote forms indicated that the deletion of TcGP72 interrupted the formation of the molecular structure of the FAZ, limiting its localization only to the flagellar pocket. In contrast, the deletion of TcFLA‐1BP did not completely interrupt the formation of the molecular structure of the FAZ, since the labeling was still observed in the cell body of the parasites, even with the flagellum detached. Two models have been proposed for the assembly of the FAZ: the “push” model, where the FAZ grows progressively with the addition of new proteins to the flagellar pocket region as the flagellum lengthens, and the “pull” model, which suggests the existence of a protein complex in the flagellum that “pulls” the FAZ into formation (Kohl et al. 1999; Sunter and Gull 2016). Our results indicate that, in the absence of the TcGP72 protein from the FAZ domain of the flagellum, there is a nucleation of proteins in the region of the flagellar pocket, but without the elongation or proper distribution of these proteins. In contrast, in the parasite knocked out for TcFLA‐1BP, FAZ distribution occurs even without the presence of this protein, although in parasites with partially detached FAZ, there is an interruption in labeling. In *T. brucei*, RNAi knockdown of the TbFLA1 and TbFAZ5 proteins in the FAZ intracellular domain (Rotureau et al. 2014; Sunter et al. 2015) also generates parasites with partially detached flagella, showing TbFAZ1 labeling in the cell body, similar to parasites knocked out for TcFLA‐1BP.

These findings reinforce the hypothesis that the FAZ substructures are preassembled and transported to the assembly site, probably in the flagellar pocket. As reported by (Sunter and Gull 2016), this is similar to what occurs in desmosomes, which pre‐form protein complexes and store them in vesicles, which are then transported to the membrane. Our data suggest that, in the absence of TcGP72, these complexes accumulate in the proximal region of the flagellum, indicating the dependence of FAZ formation on flagellar domain proteins. On the other hand, the labeling of L3B2 in TcFLA‐1BP knockout parasites supports the idea that the complexes are preassembled, but also suggests that, in the absence of TcFLA‐1BP in the flagellum, other proteins may continue to interact with the flagellum membrane.

The deletion of the TcGP72 and TcFLA‐1BP proteins not only affected the transport of proteins associated with the FAZ complex but also altered the distribution of trans‐sialidase (TS), a protein specific to trypomastigote forms, detected by the Mab39 monoclonal antibody. There is a significant accumulation of TS near the flagellar pocket, especially in the TcGP72 mutant, as well as a greater distribution along the flagellum. In both mutants, the pattern of TS labeling is distinct from that of the T7Cas9 control. These results show that the trypomastigote forms released by the null mutants express proteins specific to this stage, as also reported by (Jesus et al. 1993) when they analyzed infective forms of parasites knocked out for the TcGP72 protein. This suggests that, molecularly, these forms retain the characteristics of trypomastigotes, despite the morphological changes observed.

Trans‐sialidases represent a large family of glycoproteins with diverse functions, anchored to the membrane by GPI (glycosylphosphatidylinositol). Although most TS genes do not encode proteins with enzymatic activity, a specific subgroup is responsible for catalyzing the transfer of sialic acid from the host's cell surface to the parasite′s mucins (Cremona et al. 1999; da Fonseca et al. 2019; Schenkman et al. 1993). This process is crucial for the parasite′s pathogenicity, acting as one of the immune system′s main evasion mechanisms, facilitating invasion and the spread of infection (Freire‐de‐Lima et al. 2017). TS are synthesized in the endoplasmic reticulum (ER), transferred to the Golgi complex, and accumulated in associated vesicles that are transported to the membrane of the contractile vacuole and flagellar pocket (Niyogi et al. 2014). Parasites deficient in TS can invade cells, but their intracellular life cycle is significantly altered, with difficulties in differentiating from amastigotes to trypomastigotes and in being released from the host cell (Burle‐Caldas et al. 2022).

The results obtained with the TcGP72 and TcFLA‐1BP null mutants suggest that the proper assembly of the flagellar assembly (FAZ) structure is important for protein transport. These findings highlight the potential role of the FAZ structure in guiding the localization of its molecular components and influencing the trafficking of other proteins, such as trans‐sialidases, through the flagellar pocket. Although studies of *Leishmania mexicana* lacking LmxFAZ5 showed no significant changes in the localization of surface markers such as lipophosphoglycan (LPG), gp63 and amastins (Sunter et al. 2019). Our results indicate that the absence of TcGP72 and TcFLA‐1BP in *T. cruzi* leads to subtle but detectable changes in the pattern of TS labeling. Although these alterations are modest, they suggest that proper assembly of the FAZ structure may play a role in modulating protein trafficking.

When analyzing the pathogenicity of the parasite in the absence of TcGP72, we observed that its life cycle in vertebrate hosts closely resembled that of the control group across all evaluated parameters. The parasite′s ability to adhere to host cells, the percentage of infected cells, the number of parasites per cell, the rate of intracellular multiplication, and the release of parasites from host cells were comparable to those of the control group. These results align with the findings of (Jesus et al. 1993). However, in contrast to their study, we identified trypomastigote forms with detached flagella in our cultures, morphologically similar to those described by (Jesus et al. 1993) as mesomastigotes.

However, our results demonstrate that the disruption of TcFLA‐1BP significantly impacts the parasite′s intracellular life cycle compared to both the control and the TcGP72 null mutant. Trypomastigotes from the TcFLA‐1BP null mutant exhibited reduced adhesion to host cells, a lower infection rate, and fewer parasites per cell, which directly influenced an extended intracellular generation time and delayed the egress of trypomastigote forms. These findings suggest that the TcFLA‐1BP protein plays a pivotal role in various stages of the parasite′s life cycle, affecting its capacity to infect and replicate within host cells, in contrast to the role of TcGP72.

Previous studies on *Leishmania mexicana* demonstrated that FAZ5 deletion reduces pathogenicity in both invertebrate and vertebrate hosts (Sunter et al. 2019). Consistently, our findings in *Trypanosoma cruzi* show that TcFLA‐1BP deletion, a FAZ‐associated protein in the cell body domain of the flagellum, similarly impairs infection establishment. In *T. brucei*, FAZ integrity is even more critical, as deletion of any FAZ component is lethal (Wheeler et al. 2016). Our data indicates that FAZ deletions in *T. cruzi* differentially affect infection, with a more pronounced impact when targeting the cell body domain.

The morphology of *T. cruzi* is defined by subpellicular microtubule organization, organelle positioning, and membrane protein composition (Sinclair and de Graffenried 2019; Vidal and De Souza 2022). Flagellum, nucleus, kinetoplast positioning, and microtubule orientation determine cell shape and polarity. The FAZ acts as a structural scaffold, coordinating cytoskeletal and membrane organization for morphogenesis (Rodríguez‐Bejarano et al. 2021; Romano et al. 2012; Souza‐Melo et al. 2023; Tomasina et al. 2024; Vidal and Souza 2017). During infection, trypomastigotes undergo intracellular reorganization, including membrane fusion, organelle repositioning, and kDNA redistribution, facilitating differentiation into amastigotes. This structural remodeling is critical for adaptation to the host environment (De Souza 1999; Tomasina et al. 2024; Vidal and Souza 2017; Zuma et al. 2021).

In this study, transmission electron microscopy revealed that T7Cas9 and TcFLA‐1BP knockout lines exhibited the typical morphology of trypomastigotes, and the TcGP72 knockout mutant presented significant morphological alterations. The results indicate that the TcGP72 protein plays a crucial role in maintaining the structural integrity of the flagellar attachment zone, directly impacting intracellular organization. Disorganization of the kDNA network and displacement of organelles, such as the kinetoplast and Golgi apparatus, were evident in the mutant trypomastigotes. The nucleus of mutant parasites exhibited variable morphologies, with predominant heterochromatin and absence of nucleolus. The organization of the kDNA was also altered, with total or partial decompaction and topological redistribution of the kinetoplast. These results suggest that decompaction of the kDNA network and kinetoplast redistribution are independent processes, regulated by distinct mechanisms. The literature describes that the structural conversion of the kDNA network from a discoid to a globular shape depends on the control of kinetoplastid‐associated proteins (KAPs) or structures associated with these proteins in different forms of *T. cruzi* (Cavalcanti et al. 2009; De Souza 1984).

The reorganization of the Golgi apparatus was another significant alteration observed, suggesting that the characteristic position of this organelle, between the kinetoplast and the nucleus in T7Cas9 cells, is dependent on the integrity of the FAZ. In TcGP72 knockout mutants, the Golgi was found in a position opposite to the kinetoplast, corroborating the hypothesis that TcGP72 plays a central role in the spatial organization of organelles, indirectly, through its influence on FAZ formation. These observations are in agreement with studies that emphasize the FAZ as a fundamental structural framework for morphogenesis and cytoskeletal organization in trypanosomatids (Halliday et al. 2020).

Thus, the alterations observed in the Golgi apparatus, kinetoplast, and kDNA network of TcGP72 knockout trypomastigotes highlight the central importance of the TcGP72 protein in the spatial organization of intracellular components and in maintaining cellular architecture. As a fundamental structural component of the flagellar attachment zone (FAZ), TcGP72 appears to act as an organizer of the positions of key organelles and the structural integrity of the parasite, reinforcing the relevance of this gene in the morphogenesis and adaptation of *T. cruzi* throughout its life cycle.

Although the amastigote forms of the null mutants did not exhibit significant ultrastructural abnormalities, rare but consistent alterations were observed in TcFLA‐1BP^−/−^ parasites, including close contact between the mitochondrial and nuclear membranes, as well as the occasional presence of parasites containing more than one nucleolus. The prolonged intracellular cycle of TcFLA‐1BP‐deficient amastigotes may have enabled the identification of transient structural anomalies that are usually not evident in wild‐type parasites due to their faster developmental progression. Despite the limited scope of our ultrastructural analysis, these alterations consistently appeared in knockout parasites and were absent in control groups, suggesting a biologically meaningful association. The presence of such atypical phenotypes likely reflected downstream consequences of delayed intracellular development.

Furthermore, the FAZ is recognized as both a flagellar adhesion structure and a central cytoskeletal organizer that coordinates cell polarity, morphology, and organelle positioning (Souza‐Melo et al. 2023; Halliday et al. 2020; Sunter and Gull 2016). Disruptions in specific FAZ subdomains lead to global cytoskeletal disorganization, affecting the spatial arrangement of key intracellular structures, including the nucleus and mitochondria. The findings of this study reveal that the deletion of TcGP72 and TcFLA‐1BP affects *T. cruzi* differently throughout the parasites life cycle. These distinct phenotypic outcomes may stem from the disruption of specific FAZ subdomains, where each protein is localized. Given that the FAZ functions as a spatial organizer of the cytoskeleton, impairments in different regions of this structure likely lead to unique consequences for cell morphology, division, and host‐parasite interactions.

## 4 Experimental Procedures

### 4.1 Host Cell

LLC‐MK2 cells (Rhesus monkey kidney epithelial cells) were cultured in 25 or 125 cm^2^ culture flasks in RPMI 1640 medium (Gibco) supplemented with 10% fetal bovine serum (FBS) and 1% penicillin‐streptomycin (Gibco). Cells were maintained in a humidified incubator at 37°C with 5% CO_2_. The culture medium was replaced every 2–3 days, and cells were sub‐cultured when they reached 80% confluence.

### 4.2 Parasites Strains and Culture

The *Trypanosoma cruzi* lines used in this study were previously generated by our group (Souza‐Melo et al. 2023). The parental strain consisted of Dm28c epimastigotes engineered to express both T7 RNA polymerase and SpCas9 (T7Cas9), enabling CRISPR/Cas9‐mediated gene editing. TcFLA‐1BP^−/−^ is a double knockout line for the TcFLA‐1BP gene (ID C4B63_21g106), which encodes an FLA1‐binding protein, while TcGP72^−/−^ is a double knockout for the TcGP72 gene (ID C4B63_21g104), which encodes a flagellum adhesion protein. All parasite lines were cultivated in Liver Infusion Tryptose (LIT) medium supplemented with 10% fetal bovine serum (FBS), 10 U/mL penicillin, and 10 μg/mL streptomycin. Cultures were maintained at 28°C in the exponential growth phase by regular passages and seeding at a density of 5 × 10^6^ cells/mL (logarithmic growth phase). The T7Cas9 parental strain was cultured in LIT medium supplemented with 200 µg/mL G418, whereas the TcFLA‐1BP^−/−^ and TcGP72^−/−^ lines were maintained in medium containing 25 µg/mL blasticidin and 200 µg/mL hygromycin.

Metacyclogenesis: Epimastigotes were differentiated into metacyclic trypomastigotes in vitro using a two‐step culture system. Briefly, epimastigotes in the late exponential growth phase (~ 5−7 × 10^7^ cells/mL) in LIT medium were harvested by centrifugation and resuspended in triatomine artificial urine (TAU) medium (190 mM NaCl, 17 mM KCl, 2 mM MgCl₂, 2 mM CaCl_2_, 8 mM phosphate buffer, pH 6.0) at a density of 1 × 10^9^ cells/mL. After a 1‐h incubation at 28°C to induce stress, the parasites were transferred to 100 mL of TAU3AAG medium (TAU medium supplemented with 50 mM sodium l‐glutamate, 2 mM sodium l‐aspartato, and 10 mM glucose) in a 175 cm^2^ culture flask at a final density of 1 × 10^7^ cells/mL (Contreras et al. 1985). The culture was then incubated at 28°C for 96 h, after which metacyclic trypomastigotes were purified by DEAE cellulose chromatography (Teixeira and Yoshida 1986).

Cell culture‐derived trypomastigotes (TCTs): TCTs were obtained from infected LLC‐MK2 cell cultures. Cells were infected with purified metacyclic trypomastigotes at a multiplicity of infection (MOI) of 20:1 and incubated for 24\ h at 37°C in a 5% CO_2_ atmosphere to allow parasite internalization. After this period, the medium was replaced, and the cells were incubated for an additional 15 days. Released trypomastigotes were collected by centrifugation at 2600 g. Parasite purity and viability were assessed by light microscopy and counting in a Neubauer chamber.

### 4.3 Confirmation of Deletions in Trypomastigotes

To confirm gene deletions, trypomastigotes derived from cell culture were collected. Genomic DNA (gDNA) was extracted and used in PCR reactions with specific oligonucleotides for the TcFLA‐1BP, TcGP72, and the blasticidin and hygromycin resistance genes, as described by (Souza‐Melo et al. 2023). The primers used were: C1 FLA_1BP 5′UTR forward (TTCAGCAATAAGAAGAAGGAAGGG), C2 FLA1_BP ORF reverse (TACCTTCATAGAGCTTCACATCGG), C1 GP72 5′UTR forward (AAGAAGAGAGAGGGAGAGAGAG), C2 GP72 ORF REV (AAATGCCGGATTTGTTCACCAAAC), B1_BSD_Fow (CCTCATTGAAAGAGCAACGGC), B2_BDS_Rev (AGGGCAGCAATTCACGAATC), H1_Hygro_Fow (GAAAAAGCCTGAACTCACCGC), and H2_Hygro_Rev (GTGTCGTCCATCACAGTTTGC). PCR products were electrophoresed on a 1% agarose gel, stained with ethidium bromide, and visualized using a UVP bioimaging system.

### 4.4 Parasite‐Host Cell Interaction

Tissue culture‐derived trypomastigotes of Dm28c wild‐type, TcFLA‐1BP^−/−^ and TcGP72^−/−^ strains were obtained from the supernatant of LLC‐MK2 cells previously infected with metacyclic trypomastigotes. TCTs were cultured in RPMI medium under humidified conditions with 5% CO_2_ at 37°C. Third passage (P3) TCTs were used for all in vitro host cell assays. For cell infection, 5 × 104 LLC‐MK2 cells were seeded per well in 24‐well plates and incubated for 24 h at 37°C with 5% CO_2_. Cells were then infected with TCTs at a multiplicity of infection (MOI) of 10 parasites per cell. The TCTs were incubated with mammalian cells for 4 h in an oven with 5% CO_2_ at 37°C. For the 24‐ and 48‐h time points, the same steps were performed, but after the 4‐h incubation with TCTs, the plates were washed with serum‐free RPMI and then incubated with RPMI medium containing 5% serum for an additional 24 or 48 h in the oven with 5% CO_2_ at 37°C. To assess the initial adhesion of tissue culture‐derived trypomastigotes to LLC‐MK2 cells, a 1‐h assay was performed. LLC‐MK2 cells were infected with TCTs at a multiplicity of infection (MOI) of 20:1 and incubated for 1 h at 3°C in a 5% CO₂ atmosphere. At the specified time points, cells were fixed and stained with Giemsa. The mean number of parasite‐infected cells and the mean number of parasites per infected cell were counted in 300 cells per well in quadruplicate using light microscopy.

To determine adhesion, infection percentage, infection index, number of intracellular parasites, and multiplication rate the following calculations were applied. Adhesion (parasites attached per cell): (Number of cells with adhered parasite x Total number of parasites adhered/Total cells count)/100. Percentage of infected cells (%iC): %iC = (Number of infected cells/Total cells count) × 100. Infection index: Infection index = %iC × Number of intracellular parasites/Total number of cells. Number of intracellular parasites: Number of intracellular parasites = (Infection index × Total number of cells)/100. The multiplication rate (T) of the parasite was determined using the formula: T = time (in hours) × Log2/Log (final parasite count/initial parasite count).

Statistical analysis: Data were analyzed using one‐way ANOVA followed by Tukey′s multiple comparisons test in Prism (GraphPad Software). Statistical significance was considered at a *p* value of < 0.001 compared to the control group.

### 4.5 Light Microscopy

Cells from T7Cas9, TcFLA‐1BP^−/−^, and TcGP72^−/−^ strains (1 × 107 cells) were fixed with 4% paraformaldehyde in PBS for 10 min at room temperature. After washing PBS, cells were stained with Hoechst 33342 (3 µg/mL–Thermo Fisher) in PBS for 10 min at room temperature. Cells were then adhered to poly‐l‐lysine‐coated coverslips and mounted in ProLong Gold antifade mountant (Thermo Fisher). Samples were visualized using a Zeiss Axio Observer Z1 fluorescence microscope equipped with a 100×/1.25 oil immersion objective. Images were analyzed with ImageJ software to quantify flagellar detachment and the localization of the nucleus and kinetoplast in at least 300 cells per experiment.

Immunofluorescence: Cells from T7, TcFLA‐1BP^−/−^, and TcGP72^−/−^ strains (1 × 10⁷ cells) were fixed with 4% paraformaldehyde in PBS for 10 min at room temperature. After fixation, cells were adhered to poly‐l‐lysine‐coated coverslips. To block nonspecific binding sites, cells were incubated with 3% bovine serum albumin (BSA) in PBS for 1 h. Cells were then incubated with primary anti‐TS antibody (Mab39 kindly provided by Dr. Sergio Schenkman) diluted 1:200 in PBS containing 3% BSA for 1 h at room temperature. For the L3B2 antibody (kindly provided by Dr. Keith Gull), the same protocol was used, except that after fixation, cells were permeabilized with 0.1% Triton X‐100 for 5 min followed by a 1‐h block with 3% BSA. After washing PBS, cells were incubated with Alexa Fluor 546‐conjugated secondary antibody (Invitrogen) diluted 1:500 in PBS containing 3% BSA for 1 h. Nuclei and kinetoplasts were stained with Hoechst 33342 (3 µg/mL–Thermo Fisher) for 10 min. Slides were mounted with ProLong Gold antifade mountant (Thermo Fisher) and visualized using a Zeiss Axio Observer Z1 fluorescence microscope equipped with a 100×/1.25 oil immersion objective. Images were acquired and analyzed using Zen software.

### 4.6 Ultrastructural Analysis

For scanning electron microscopy (SEM) analyses, cells were fixed for 1 h in 2.5% glutaraldehyde diluted in cacodylate buffer (0.1 M and pH 7.2). They were then adhered to coverslips, precoated with poly‐l‐lysine, and post‐fixed for 1 h with 1% osmium tetroxide diluted in cacodylate buffer. Samples were dehydrated in a graded ethanol series (50%, 70%, 90%, and two exchanges of 100% ethanol for 10 min each step), and critical point dried using CO_2_. Specimens were coated with a 5 nm layer of platinum and then visualized in an EVO 10 and ZEISS FIB‐SEM AURIGA 40 scanning electron microscope at the multiuser unit of the National Center for Structural Biology and Bioimaging (CENABIO) at UFRJ. Cell lengths were measured in the SEM images using the Axio‐Vision4 program.

For transmission electron microscopy (TEM), parasites were washed twice in PBS and fixed in 2.5% w/v glutaraldehyde in 0.1 M cacodylate buffer at pH 7.2 for 1 h. Then, cells were washed in 0.1 M cacodylate buffer (pH 7.2) and postfixed for 1 h using an osmium‐thiocarbohydrazide‐osmium (OTO) protocol ((Alcantara et al. 2018; Willingham and Rutherford 1984). After postfixation, samples were washed in water, dehydrated in a series of increasing acetone concentrations, and embedded in epoxy resin. Ultrathin sections were obtained using an Ultracut Reichert Ultramicrotome and mounted on 400‐mesh copper grids. Samples were stained with uranyl acetate and lead citrate and then analyzed using a Hitachi HT 7800 and a FEI spirit model transmission electron microscope operating at 100 and 120 kV, respectively, at the National Bioimaging Center (CENABIO) located at the UFRJ.

## Author Contributions

**Normanda Souza Melo:** conceptualization, data curation, formal analysis, investigation, visualization, methodology, validation, writing – original draft. **Giovanna Henriques de Souza:** investigation, visualization. **Wanderley de Souza:** conceptualization, resources, data curation, supervision, writing – review and editing.

## Supporting information

Figure S1 Revised.

Figure S2 Revised V3.

Figure S3 Revised.

Figure S4 Revised.

Figure S5 Revised.

**Figure S1:** Confirmation of TcFLA‐1BP and TcGP72 gene deletions in trypomastigote forms. **Figure S2:** Localization of Trans‐Sialidase in *Trypanosoma cruzi* Trypomastigotes via Mab39 Immunofluorescence. **Figure S3:** Adhesion assay of *Trypanosoma cruzi* trypomastigotes to LLC‐MK2 cells. **Figure S4:** Intracellular amastigote multiplication rate of *Trypanosoma cruzi*. The bar graph represents the multiplication rate of intracellular amastigotes of *T. cruzi* in T7Cas9 (control), TcFLA‐1BP^−/−^, and TcGP72^−/−^ strains after 24 and 48 hours of infection in LLC‐MK2 cells. **Figure S5:** Ultrastructural analysis of *Trypanosoma cruzi* intracellular amastigotes from TcFLA‐1BP and TcGP72 knockout mutants. Transmission electron microscopy (TEM) images of *Trypanosoma cruzi* intracellular amastigotes 48 hours after infection.

Ethical Compliance Statement.

## Data Availability

The data that supports the findings of this study are available in the supplementary material of this article.
