# Supplementary figures and images for: Effects of TcFLA‐1BP and TcGP72 Deletion on the Infectivity and Survival of Trypanosoma cruzi in Cell Cultures
Source: Cell Biol Int. 2025 Sep 3;49(11):1395–410. doi: 10.1002/cbin.70076 (PMC12519925; doi:10.1002/cbin.70076)

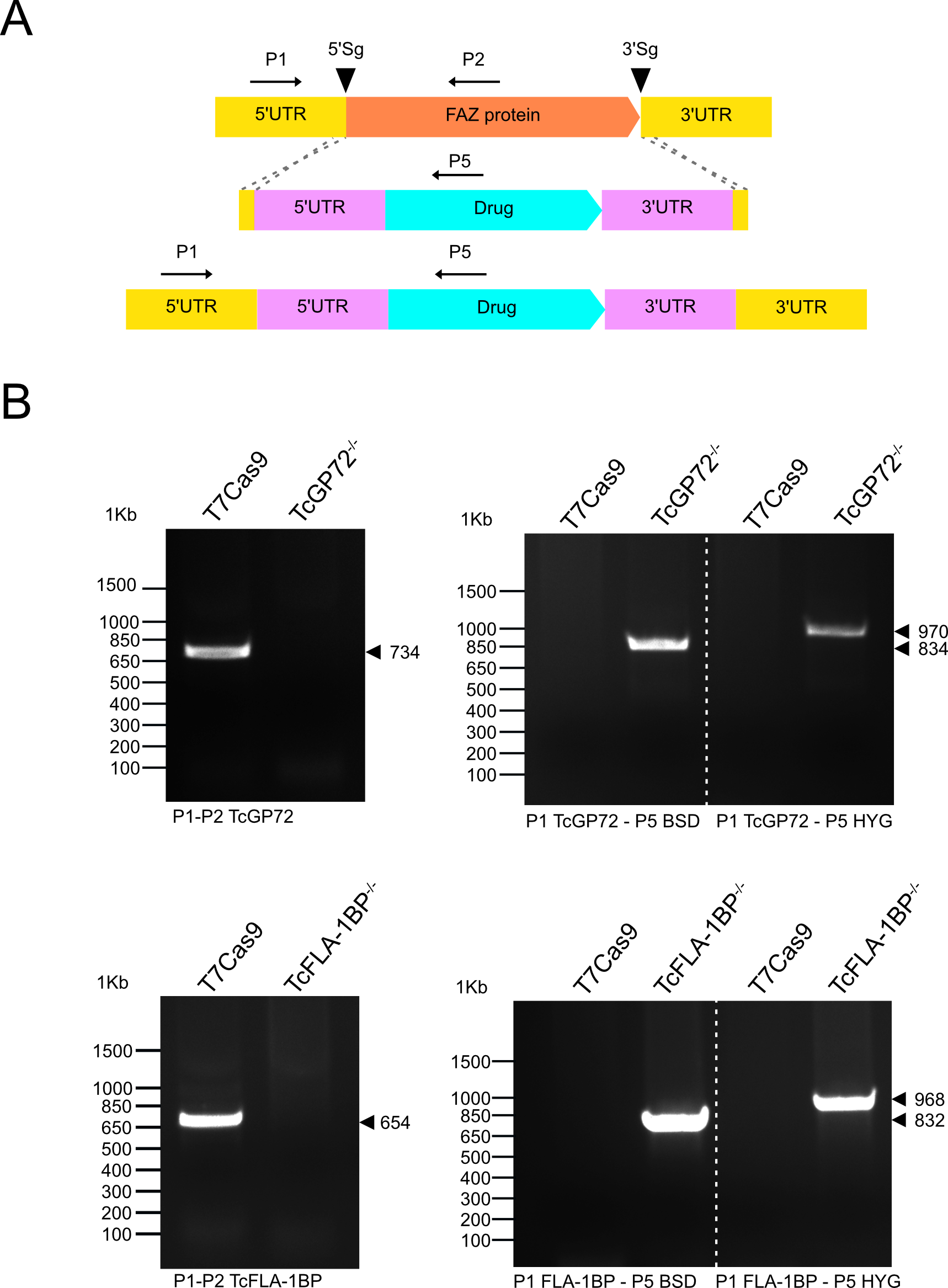

Supplement: Supplementary file 1 — Figure S1 Revised. [file CBIN-49-1395-s001.tiff]

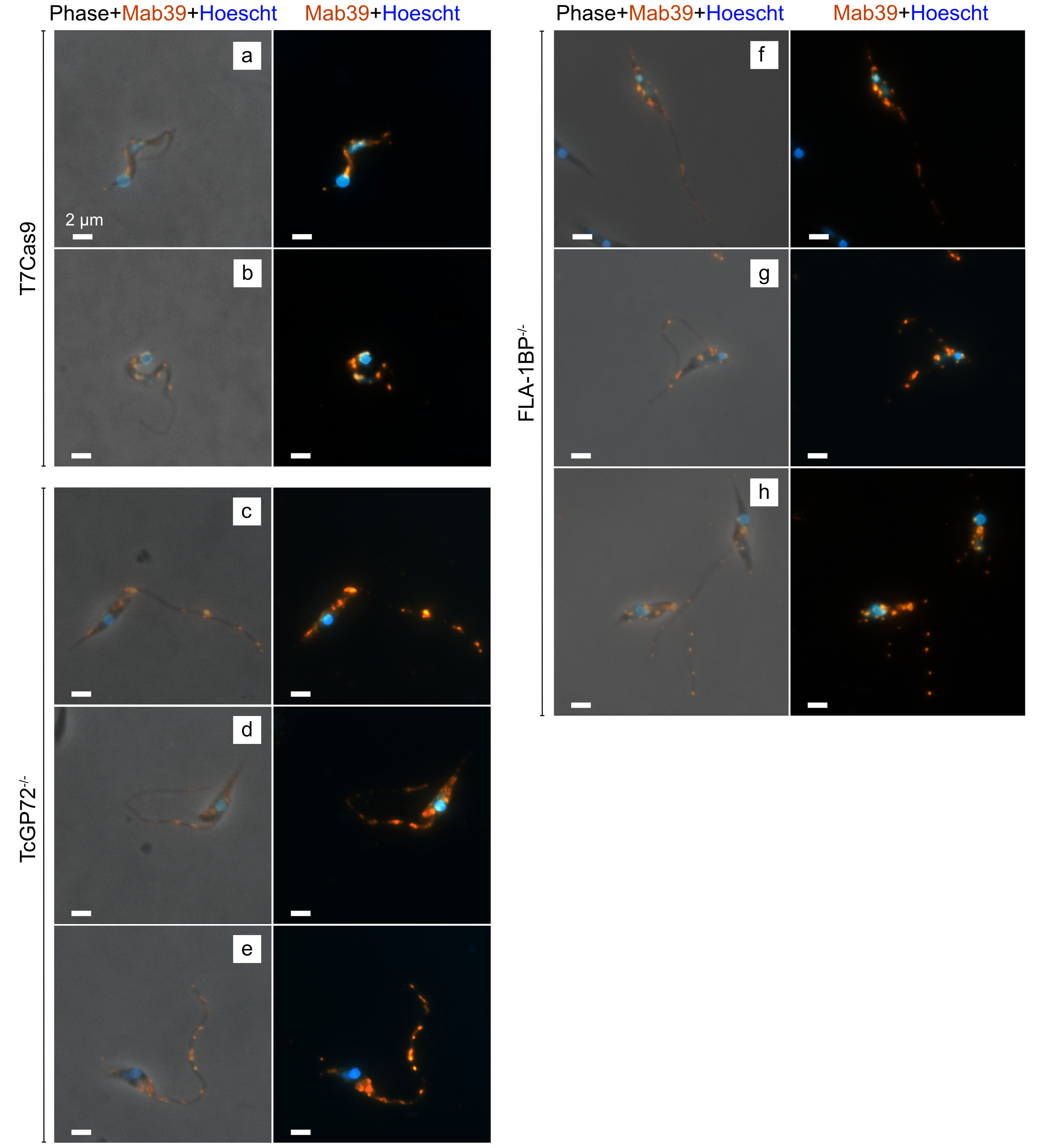

Supplement: Supplementary file 2 — Figure S2 Revised V3. [file CBIN-49-1395-s002.tiff]

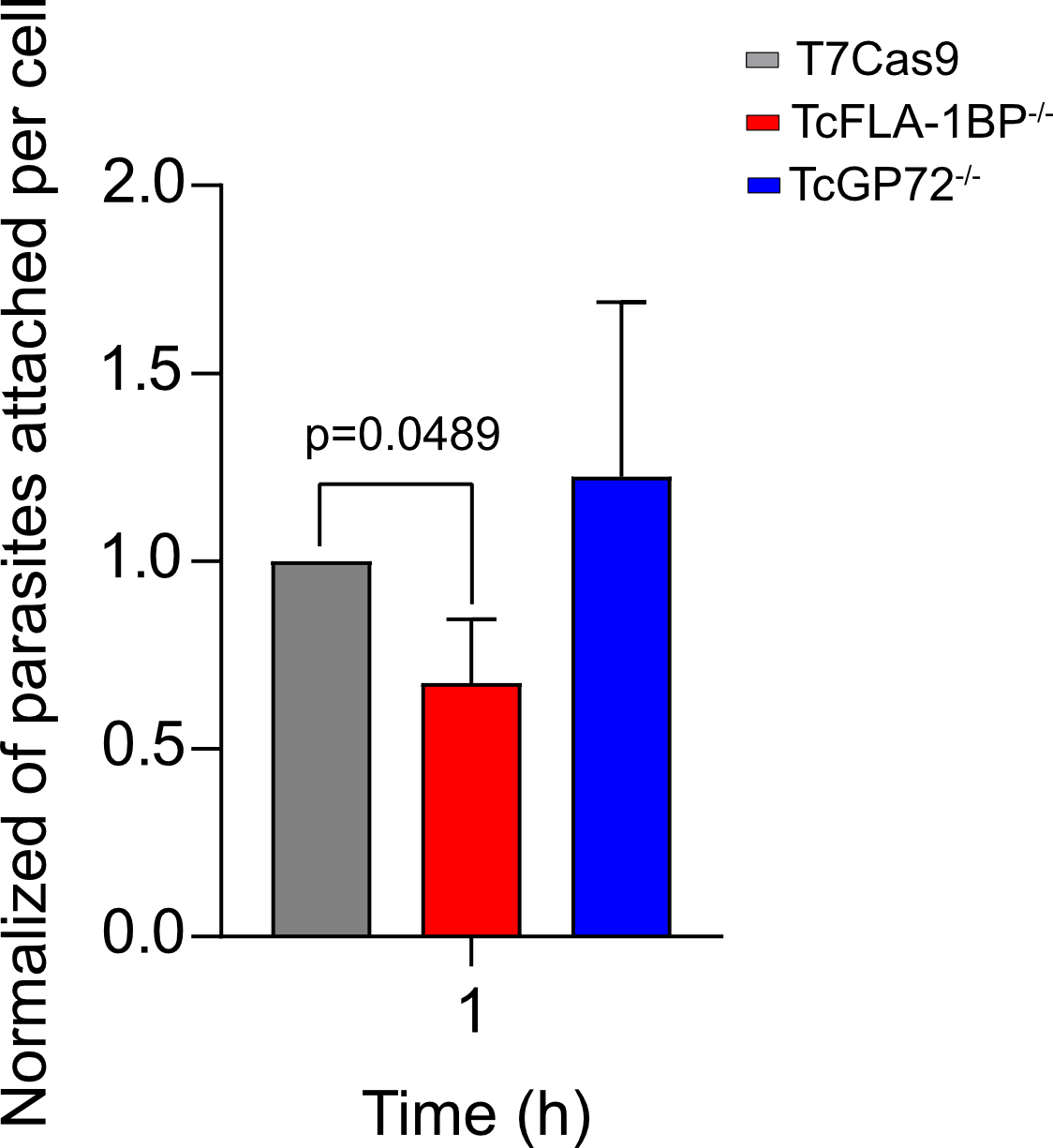

Supplement: Supplementary file 3 — Figure S3 Revised. [file CBIN-49-1395-s005.tiff]

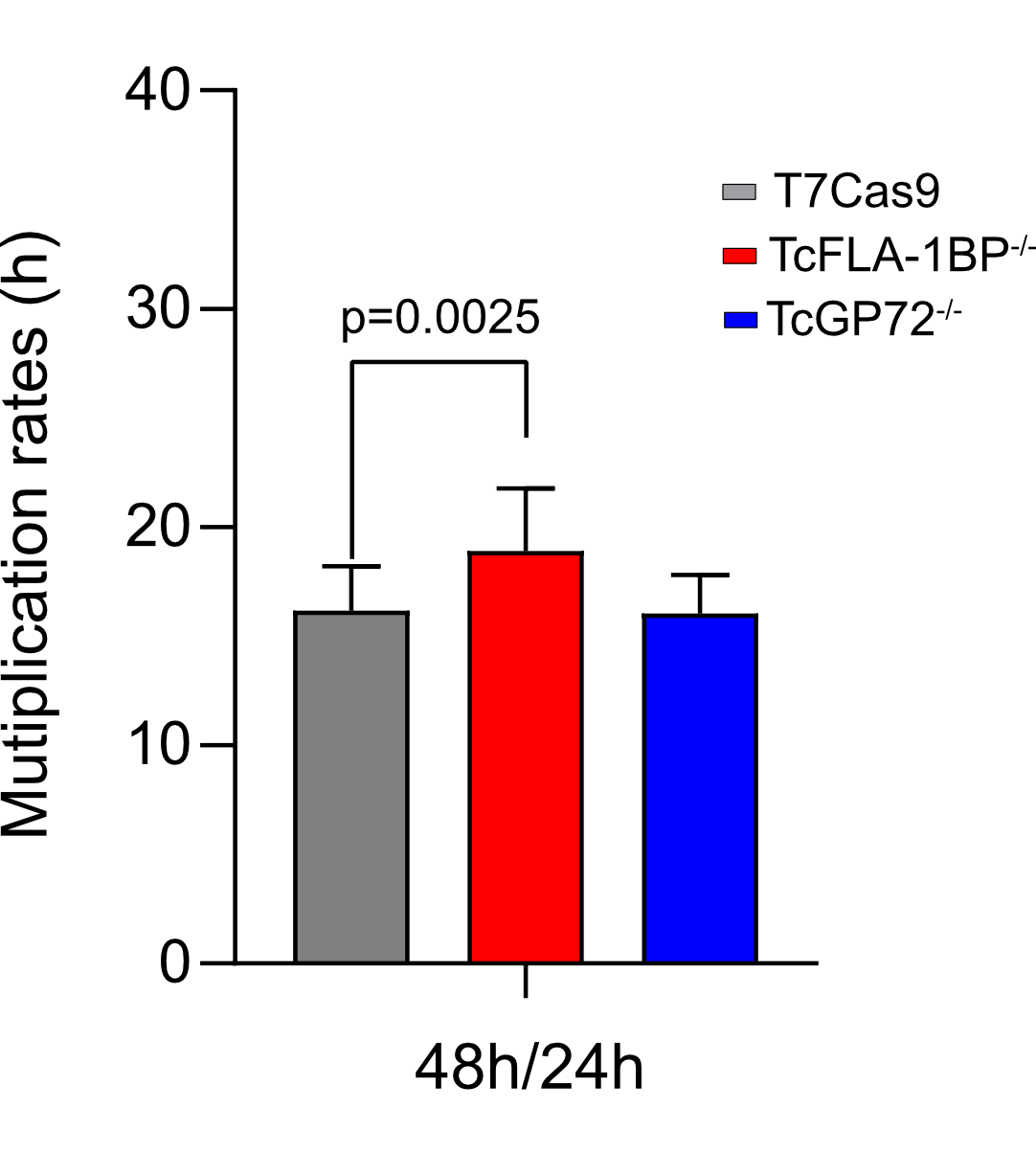

Supplement: Supplementary file 4 — Figure S4 Revised. [file CBIN-49-1395-s003.tiff]

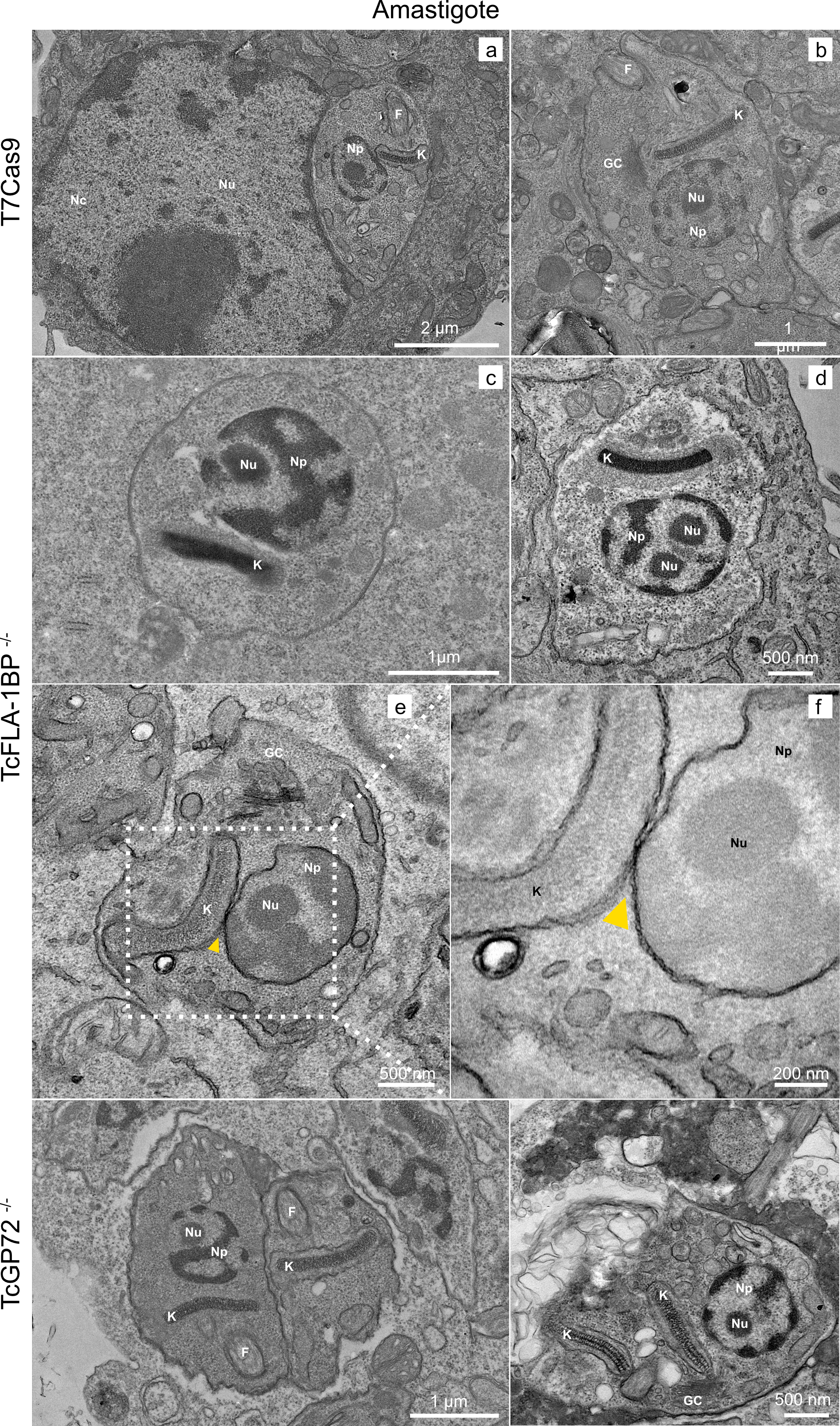

Supplement: Supplementary file 5 — Figure S5 Revised. [file CBIN-49-1395-s004.tiff]
