# Supplementary material for: Effects of TcFLA‐1BP and TcGP72 Deletion on the Infectivity and Survival of Trypanosoma cruzi in Cell Cultures
Source: Cell Biol Int. 2025 Sep 3;49(11):1395–410. doi: 10.1002/cbin.70076 (PMC12519925; doi:10.1002/cbin.70076)
Supplement: Supplementary file 6 — Figure S1: Confirmation of TcFLA‐1BP and TcGP72 gene deletions in trypomastigote forms. Figure S2: Localization of Trans‐Sialidase in Trypanosoma cruzi Trypomastigotes via Mab39 Immunofluorescence. Figure S3: Adhesion assay of Trypanosoma cruzi trypomastigotes to LLC‐MK2 cells. Figure S4: Intracellular amastigote multiplication rate of Trypanosoma cruzi. The bar graph represents the multiplication rate of intracellular amastigotes of T. cruzi in T7Cas9 (control), TcFLA‐1BP−/−, and TcGP72−/− strains after 24 and 48 hours of infection in LLC‐MK2 cells. Figure S5: Ultrastructural analysis of Trypanosoma cruzi intracellular amastigotes from TcFLA‐1BP and TcGP72 knockout mutants. Transmission electron microscopy (TEM) images of Trypanosoma cruzi intracellular amastigotes 48 hours after infection. [file CBIN-49-1395-s006.docx]

**Legend to supplementary figures**

Figure S1: Confirmation of TcFLA-1BP and TcGP72 gene deletions in trypomastigote forms. A) Schematic representation of the TcFLA-1BP and TcGP72 genes, including their 5' and 3' untranslated regions (UTRs, yellow), coding regions (TcGP72 or TcFLA-1BP, orange), and drug resistance cassettes (blasticidin-BSD or hygromycin-HYG, blue). Primers P1-P2 were used to amplify the coding regions of the genes, while primers P1-P5 amplified regions containing the integration sites of the cassettes. B) Agarose gel electrophoresis of PCR products amplified from T7Cas9 and mutant (TcGP72^-/-^ and TcFLA-1BP^-/-^) trypomastigotes. The expected band sizes for amplification with P1-P2 for TcGP72 and TcFLA-1BP are 734 bp and 654 bp, respectively. The presence of bands of 834 bp and 970 bp for TcGP72^-/-^, and 832 bp and 968 bp for TcFLA-1BP^-/-^, corresponding to the PCR products with the P1-P5 primers and the resistance cassettes (BSD and HYG), indicates the correct integration of the cassettes into the deletion sites.

Figure S2: Localization of Trans-Sialidase in *Trypanosoma cruzi* Trypomastigotes via Mab39 Immunofluorescence. Immunofluorescence assay of *Trypanosoma cruzi* trypomastigotes using the monoclonal antibody Mab39, which targets the parasite's trans-sialidase (orange). The images show the localization of trans-sialidase on the surface of trypomastigotes. Hoescht (blue) was used as a nuclear and kinetoplast stain. Panels display the distribution of trans-sialidase and the position of the nucleus and kinetoplast. T7Cas9 (control): a-b; TcGP72^-/-^: c-e and TcFLA-1BP^-/-^: f-h. Scale bars = 2 µm.

Figure S3: Adhesion assay of *Trypanosoma cruzi* trypomastigotes to LLC-MK2 cells. The bar graph shows the normalized number of parasites attached per cell for the control T7Cas9 strain (gray), TcFLA-1BP^-/-^ knockout (red), and TcGP72^-/-^ knockout (blue). Data represents the mean ± standard deviation from three independent experiments. Statistical significance was determined using one-way ANOVA with Tukey's multiple comparisons test. p < 0.001 vs. control. Total number of cells = 300.

Figure S4: Intracellular amastigote multiplication rate of *Trypanosoma cruzi*. The bar graph represents the multiplication rate of intracellular amastigotes of *T. cruzi* in T7Cas9 (control), TcFLA-1BP^-/-^, and TcGP72^-/-^ strains after 24 and 48 hours of infection in LLC-MK2 cells. Data represents the mean ± standard deviation from four independent experiments. The multiplication rate (T) = time (in hours) x Log2 / Log (final parasite count / initial parasite count). Statistical significance was determined using one-way ANOVA with Tukey's multiple comparisons test. p < 0.001 vs. control.

Figure S5: Ultrastructural analysis of *Trypanosoma cruzi* intracellular amastigotes from TcFLA-1BP and TcGP72 knockout mutants. Transmission electron microscopy (TEM) images of *Trypanosoma cruzi* intracellular amastigotes 48 hours after infection. (a-c) T7Cas9 (control); (d-f) TcFLA-1BP^-/-^, and (g-i) TcGP72^-/-^. F: Flagellum; GC: Golgi complex; K: Kinetoplast; Np: Parasite nucleus; Nc: cell nucleus; Nu: Nucleolus and yellow arrowhead: membrane contacts.
