# Supplementary material for: Effects of TcFLA‐1BP and TcGP72 Deletion on the Infectivity and Survival of Trypanosoma cruzi in Cell Cultures
Source: Cell Biol Int. 2025 Sep 3;49(11):1395–410. doi: 10.1002/cbin.70076 (PMC12519925; doi:10.1002/cbin.70076)
Supplement: Supplementary file 7 — Ethical Compliance Statement. [file CBIN-49-1395-s007.pdf]

Ethical Compliance Statement

This manuscript is a retrospective case report that does not require ethics committee approval according to the guidelines and regulations of our institution.

Sincerely,

Dr. Prof. Wanderley de Souza and Dr. Normanda Souza Melo
Federal University of Rio de Janeiro

Correspondence: normandasouzamelo@gmail.com (N.S.M. Normanda Souza-Melo); wsouza@biof.ufrj.br (W.d.S. Wanderley de Souza)
